# Mechanisms Mediating the Regulation of Peroxisomal Fatty Acid Beta-Oxidation by PPARα

**DOI:** 10.3390/ijms22168969

**Published:** 2021-08-20

**Authors:** Mounia Tahri-Joutey, Pierre Andreoletti, Sailesh Surapureddi, Boubker Nasser, Mustapha Cherkaoui-Malki, Norbert Latruffe

**Affiliations:** 1 Bio-PeroxIL Laboratory, University of Bourgogne Franche-Comté, 21000 Dijon, France; mouniajoutey@gmail.com (M.T.-J.); pierre.andreoletti@u-bourgogne.fr (P.A.); malki@u-bourgogne.fr (M.C.-M.); 2 Laboratory of Biochemistry, Neurosciences, Natural Resources and Environment, Faculty of Sciences & Techniques, University Hassan I, BP 577, 26000 Settat, Morocco; boubker.nasser@uhp.ac.ma; 3 Office of Pollution Prevention and Toxics, United States Environmental Protection Agency, Washington, DC 20460, USA; surapureddi.sailesh@epa.gov

**Keywords:** PPARα, peroxisome, β-oxidation, PPRE, ligand, coregulator, micronutrients, PPARα knockout

## Abstract

In mammalian cells, two cellular organelles, mitochondria and peroxisomes, share the ability to degrade fatty acid chains. Although each organelle harbors its own fatty acid β-oxidation pathway, a distinct mitochondrial system feeds the oxidative phosphorylation pathway for ATP synthesis. At the same time, the peroxisomal β-oxidation pathway participates in cellular thermogenesis. A scientific milestone in 1965 helped discover the hepatomegaly effect in rat liver by clofibrate, subsequently identified as a peroxisome proliferator in rodents and an activator of the peroxisomal fatty acid β-oxidation pathway. These peroxisome proliferators were later identified as activating ligands of Peroxisome Proliferator-Activated Receptor α (PPARα), cloned in 1990. The ligand-activated heterodimer PPARα/RXRα recognizes a DNA sequence, called PPRE (Peroxisome Proliferator Response Element), corresponding to two half-consensus hexanucleotide motifs, AGGTCA, separated by one nucleotide. Accordingly, the assembled complex containing PPRE/PPARα/RXRα/ligands/Coregulators controls the expression of the genes involved in liver peroxisomal fatty acid β-oxidation. This review mobilizes a considerable number of findings that discuss miscellaneous axes, covering the detailed expression pattern of PPARα in species and tissues, the lessons from several PPARα KO mouse models and the modulation of PPARα function by dietary micronutrients.

## 1. Introduction

As reported in the review by Latruffe and Vamecq [1], peroxisomes are ubiquitous, single membrane-bound organelles. They belong to the fundamental class of intracellular compartments named microbodies. According to the evolutionists, microbodies and eukaryotic cells appeared on Earth around 1.5 billion years ago. Based on their related cell origin, these organelles are defined as glycosomes, glyoxysomes, hydrogenosomes or peroxisomes. Peroxisomes are found in higher vertebrates; glycosomes exist only in trypanosomes; glyoxysomes are found in leaves and seeds; hydrogenosomes are found in anaerobic unicellular ciliates, flagellates, and fungi. The latter two microbody structures belong to lower eukaryotic species, and all these compartments metabolize hydrogen peroxide. According to the endosymbiotic theory, peroxisomes, mitochondria, and chloroplasts may have derived from free-living prokaryotic ancestors. Only mitochondria and chloroplasts are semi-autonomous organelles, containing a DNA genome, which encodes for just some of their proteins.

In the mammalian liver, very-long-chain-fatty acids (VLCFA) are exclusively shortened in peroxisome through a specific β-oxidation system. Then, shortened fatty acids are metabolized by mitochondrial β-oxidation. Peroxisomes also contain the first enzymatic steps of plasmalogen synthesis [1]. In addition, they are involved in maintaining a redox state through the NAD^+^/NADH balance, linked to the pyruvate/lactate level. In 1965, a milestone was reached by Hess et al. [2] who described, for the first time, hepatomegaly induced by clofibrate (ethyl 2-(4-chlorophenoxy)-2-methylpropanoate) in rats, subsequently established as a peroxisome proliferator in rodents and an activator of the fatty acid peroxisomal β-oxidation [3]. Later, Isseman and Green [4] identified peroxisome proliferators (PPs) as activator ligands of a special class of nuclear receptors termed Peroxisome Proliferator-Activated Receptor α (PPAR α). Afterward, several PPAR isoforms were characterized as members of the superfamily of the nuclear steroid receptors. It is recognized that the phylogenetic origin of PPARs dates back 200 million years to the fish-mammalian divergence period [5]. PPARs evolved three times faster than other members of the hormone nuclear receptor superfamily, and are represented now in three isoforms (α, β/δ, and γ).

## 2. Peroxisomal β-Oxidation Systems

**Figure 1:**
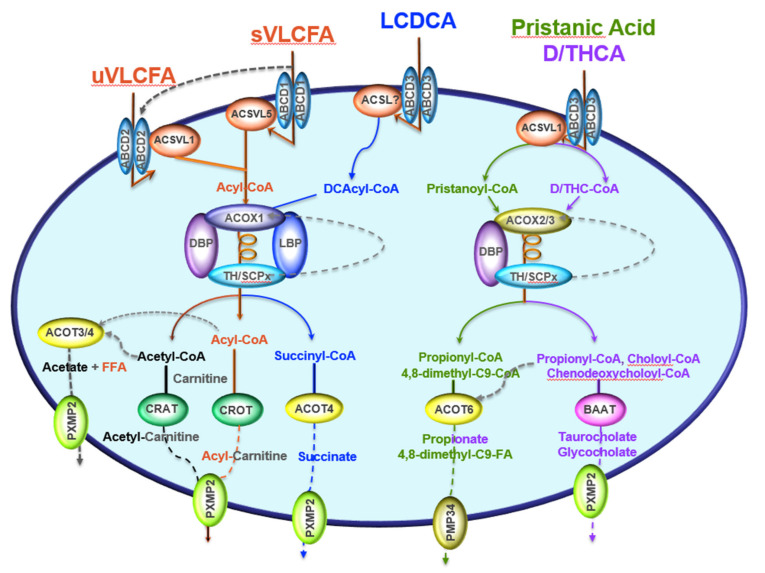
Peroxisomal β-oxidation pathways, including different enzymes and transporters. Saturated or unsaturated very-long-chain fatty acids (sVLCFA or uVLCFA) are imported by the solute ABC-transporters ABCD1 and ABCD2, respectively, into peroxisome, where they are transformed into their acyl-CoAs by one of the peroxisomal acyl-CoA synthetases (ACSVL5 for uVLCFA, and ACSVL1 for uVLCFA). The long-chain dicarboxylic acids (LCDCA), originating from the endoplasmic reticulum ω-oxidation, are imported by ABCD3 and activated to their acyl-CoA thioesters (DCAcyl-CoA) by an unknown acyl-CoA synthetase (ACSL?). The reactions that are catalyzed by ACSVL and ACSL use CoASH and hydrolyze ATP to AMP and pyrophosphate to activate VLCFA or LCDCA molecules, giving acyl-CoA. Acyl-CoA oxidase 1 (ACOX1) is the first flavoenzyme in the straight-chain β-oxidation system, oxidizing sVLCFA, uVLCFA, or LCDCA to their enoyl-CoA derivatives. The second enzyme metabolizing sVLCFA and uVLCFA is the D-bifunctional protein (also called MFP2 or HSD4B17), while the dicarboxylic enoyl-CoA are taken by the L-bifunctional enzyme (also called MFP1 or EHHADH). The thiolytic cleavage is catalyzed by one of the two peroxisomal thiolases (TH: thiolase/ACAA1/2 or SCPx: sterol carrier protein-x). After several rounds of β-oxidation, the peroxisomal system gives shortened acyl-CoA derivatives as hexanoyl- or octanoyl-CoA, and one molecule of acetyl-CoA/round. Both shortened acyl-CoA and acetyl-CoA can be hydrolyzed by acyl-CoA thioesterases 3 or 4 (ACOT3/4) to CoASH, while free fatty acid and acetate are exported by the pore-forming protein PXMP2 (or PMP22) to the cytosol. However, acetyl-CoA and acyl-CoA derivatives can also be transformed to acetyl-carnitine or acyl-carnitine by carnitine acetyl- and carnitine octanoyl transferases (CRAT and CROT), respectively, and then exported by PXMP2 to the cytosol. β-oxidation of DCAcyl-CoAs leads to the production of succinyl-CoA, hydrolyzed to succinate and CoASH by ACOT4, and shipped outside by peroxisome PXMP2. Bile acid intermediates, dihydroxycholestanoic acid (DHCA) and trihydroxycholestanoic acid (THCA), imported by ABCD3 transporter, are beta-oxidized by ACOX2, DBP and SCPx enzymes, leading to the formation of choloyl-CoA and chenodeoxycholoyl-CoA, which are conjugated to glycine or taurine by the bile acid-CoA: amino acid N- acyltransferase (BAAT) and then exported by PXMP2. D/THC-CoA indicate DHCA and THCA co-enzyme A thioesters.

In mammalian cells, both mitochondria and peroxisomes can degrade fatty acid chains. Although each organelle harbors its own fatty acid β-oxidation pathway, only the distinct mitochondrial β-oxidation system feeds the oxidative phosphorylation pathway for ATP synthesis, while the peroxisomal β-oxidation pathway participates in cellular thermogenesis [6]. Historically, we owe the first description of the mammalian peroxisomal fatty acid β-oxidation system to Lazarow and de Duve (1976) [7]. Later, a second peroxisomal β-oxidation system was characterized [6]. However, the very-long-chain fatty acids, part of the long-chain class and long-chain dicarboxylic acids, are exclusively processed by the peroxisomal β-oxidation system, whereas other common long-chain fatty acids are oxidized by mitochondria [6,8]. The entry of fatty acids into peroxisome, and activation as acyl-CoAs, depend on ABC membrane transporters (ABCD subfamily) and very-long-chain acyl-CoA synthetases [9,10]. The first β-oxidation system comprises three enzymes: acyl-CoA oxidase 1 (ACOX1), multifunctional protein (L-bifunctional peroxisomal enzyme (L-PBE, also referred to as EHHADH or MFP-1) [11,12], and 3-ketoacyl-CoA thiolases [13] (Figure 1). These three enzymes catalyze four successive reactions, starting with the α,β-dehydrogenation by ACOX1 of the acyl-CoA into 2-trans-enoyl-CoA. L-PBE catalyzes enoyl-CoA hydration into L-3-hydroxacyl-CoA, which is dehydrogenated, giving the 3-ketoacyl-CoA. Then, the 3-ketoacyl-CoA is subjected to a thiolytic cleavage by thiolase to produce one acetyl-CoA molecule and a two-carbon-shortened acyl-CoA [13] (Figure 1).

The second peroxisomal β-oxidation system (Figure 1), converting fatty carboxylates with a 2-methyl branch, such as pristanic acid and bile acid intermediates, includes the 2-methylacyl-CoA-specific oxidases (trihydroxycoprostanoyl-CoA oxidase and pristanoyl-CoA oxidase), the second multifunctional protein (named MFP-2) [11,14,15], and a 58 kDa sterol-carrier protein (SCP-2) containing thiolase activity [6,16] (Figure 1). Although both LBP/MFP-1 and DBP/MFP-2 provide hydratase and dehydrogenase activities, these proteins exhibit opposite stereospecificities. While LBP/MFP-1 hydrates 2-trans-enoyl-CoAs into L-3-hydroxyacyl-CoAs, and dehydrogenates the L-isomers [11,12], the DBP/MFP-2 transforms 2-trans-enoyl-CoAs into D-3-hydroxyacyl-CoA and dehydrogenates the D-isomers [6,11,14,15]. Despite the fact that the MFP enzymes are structurally unrelated to each other, both MFPs can hydrate 2-methyl-enoyl-CoAs [14]. The 3-hydroxy isomers formed by MFP-2 have the same (3R, 2R) configuration, or (24R, 25R) configuration in bile acid intermediates, underlining the role of MFP-2 in both pristanic acid degradation and bile acid synthesis [15]. Recently, it was demonstrated that LBP/MFP-1 is indispensable for the β-oxidation of dicarboxylic acids and the production of their medium-chain derivatives [6,15,17,18].

Distinct carnitine transferases and thioesterase enzymes handle products created from the peroxisomal β-oxidation fatty acyl-CoA derivatives. Carnitine moiety is then transferred to the acyl-CoA or the acetyl-CoA by carnitine octanoyltransferase (CROT) or carnitine acetyltransferase (CRAT). On the other hand, a specific peroxisomal thioesterase can hydrolyze the acyl-CoA or the acetyl-CoA, giving a free fatty acid or acetate that can be transported to the cytosol by the peroxisomal membrane solute transporters, such as PXMP2 or PMP34 (Figure 1) [19].

## 3. Peroxisome Proliferator Response Element, PPRE

**Figure 2:**
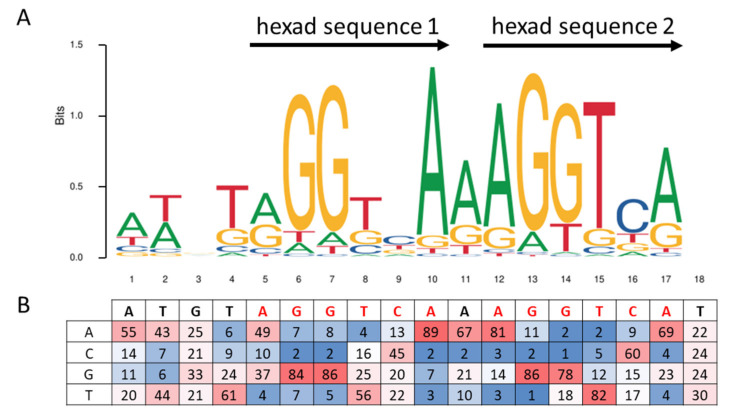
Sequence logo and consensus matrix of the PPARα/RXRα PPRE consensus sequence from MA1148.1 Jaspar matrix [38]. (**A**) Sequence logo of the MA1148.1 Jaspar matrix, presenting the conservation of nucleotides from multiple alignments of 1000 PPARα/RXRα PPRE sequences. Adenosine (A), cytidine (C), guanosine (G), and thymidine (T) nucleotides are respectively green-, blue-, yellow-, and red-colored, and the relative size of the letters represents their frequency in the consensus. The total height of a logo position corresponds to the degree of conservation in the corresponding multiple sequence alignment. (**B**) A table representing the percentage of the four bases for each position of the consensus. The color gradient code highlights the percentage of conservation of bases from blue to red for the whole table.

PPARα is an ultimate lipid sensor [20] that has the potential to orchestrate and prompt the expression of a plethora of target genes implicated in a broad range of fatty acid metabolism processes [21,22], particularly under conditions of fasting-induced lipolysis and a lipid-rich diet [23,24,25]. Indeed, PPARα activates many enzymatic pathways involved in fatty acid uptake, intracellular transport [26,27], fatty acid activation and β-oxidation, lipogenesis, ketogenesis and lipoprotein/cholesterol metabolism [28]. As a member of the PPARs family, PPARα regulates the target gene expression in a transcriptional manner through heterodimerization with another transcription factor, the retinoid X receptor (RXR) encoded by the *NR2B1* gene [29,30]. Once activated by a ligand in the ligand-binding domain (LBD), the dimer binds to a specific DNA sequence element, the peroxisome proliferator response element (PPRE), located in the promoter region of target genes, to modulate their expression [31]. It is noteworthy that this regulation can require the recruitment of coregulators [32,33,34,35,36]. The PPARα response element is usually composed of a direct repeat 1 type (DR-1), which means two immediate repetitions of the hexanucleotide AGGTCA consensus sequence, spaced by one nucleotide [37] (Figure 2). PPARα and RXRα bind the first and the second hexamer sequences, respectively. The sequence logo of the PPARα/RXRα PPRE consensus sequence ATGTAGGTCA**A**AGGTCA from the MA1148.1 Jaspar matrix [38], and the associated percentage of the four nucleotides at each position, is presented in Figure 2.

**Figure 3:**
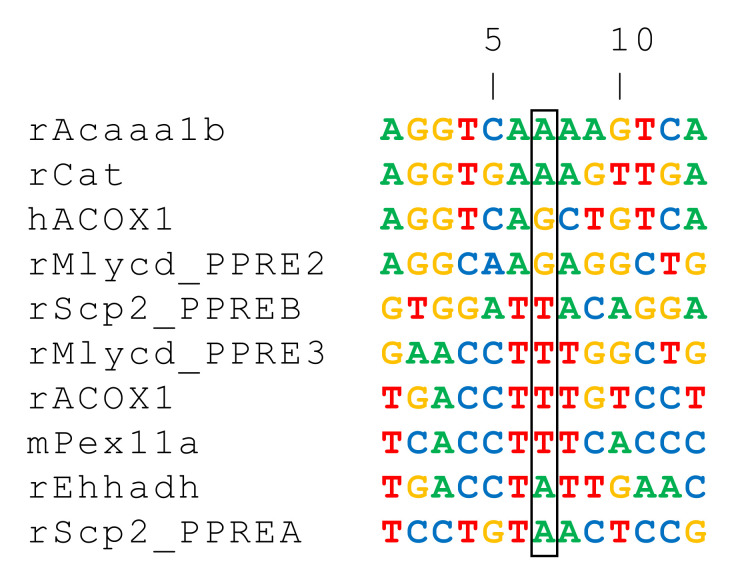
Multiple alignments of PPRE sequences, identified in peroxisomal gene promoters, and experimentally proved to be regulated through PPARα binding. Adenosine (A), cytidine (C), guanosine (G) and thymidine (T) nucleotides are respectively green-, blue-, yellow- and red-colored.

Among the hundreds of genes known to be regulated by PPARα, eight are encoding enzymes that are commonly localized in the peroxisomal compartment (Figure 3), and belong to the three species: human, mouse, and rat. Table 1 presents validated PPREs sequences for functional genes, of which four (*Acox1*, *Ehhadh*, *Acaa1b*, and *Scp2*) are encoding very-long-chain fatty acid β-oxidation enzymes, and the remaining genes are *Cat*, encoding the catalase enzyme, and *Mlycd* (malonyl-CoA decarboxylase gene), expressing an enzyme with both cytoplasmic and peroxisomal localization. The latter form is believed to be involved in the peroxisomal degradation of malonyl-CoA produced by odd-chain-length dicarboxylic fatty acid β-oxidation [39], and finally, the *Pex11a* gene, which participates particularly in peroxisome biogenesis (Pex11α).

## 4. PPARs and PPARα Structure and Function

Peroxisome proliferator-activated receptors (PPARs) are ligand-regulated transcription factors and belong to a nuclear steroid/thyroid hormone receptor superfamily [48]. Their name originates from their property of peroxisome proliferation [49]. Three PPAR isoforms have been first isolated from the mouse [4], then Xenopus [33,50,51], then rat [52] and human [53], including PPARα (NR1C1), PPARβ/δ (NUC1, NR1C2) and PPARγ (NR1C3). Human PPARα protein consists of 468 amino acid residues, while PPARβ/δ has 441, and PPARγ, 479 aminoacyls long [54]. Each is characterized by a distinct tissue expression profile, a definite ligand binding specificity, and a set of functions implicated in carbohydrate-lipid metabolism, cancer, inflammation, cell proliferation, and differentiation [55,56,57,58]. To sustain their protein stability and transcriptional activity, PPARs are subjected to post-translational modifications, such as phosphorylation [59], SUMOylation [60], and ubiquitylation [61]. PPARs act altogether in harmony, to maintain and control cellular and whole-body energy homeostasis by modulating the expression of their specific target genes [57].

**Figure 4:**
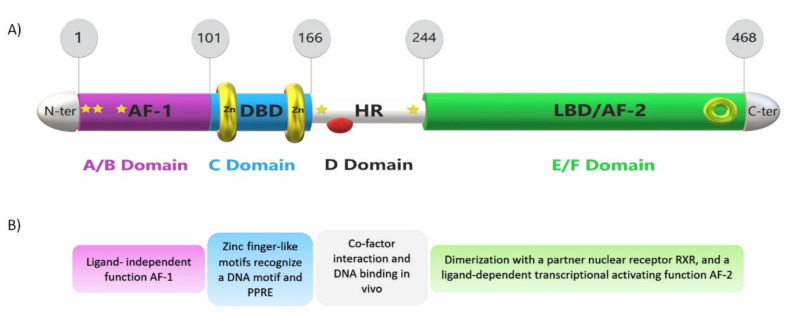
Schematic view of PPARα structure and domain function, with phosphorylation and cofactor binding sites. From the left N-terminus to the right C-terminus of the PPARα protein, (**A**) domain structure of the PPARα protein with the ligand-independent activation function-1 (AF-1) domain or A/B domain shown in purple, the DNA-binding domain (DBD) or C domain shown in blue with two zinc finger-like motifs, the hinge region (HR) or D domain shown in white, and the ligand-binding domain (LBD) or E domain together with the activation function 2 (AF-2) or F domain shown in green. Phosphorylation sites are labeled with yellow stars (6, 12, 21, 179, 230) amino acids, the corepressor site is marked with a red half-sphere, and the coactivator binding site is shown with a green ring. The panels on top show the number of amino acid residues. (**B**) Structural function of A/B, C, D, and E/F domains, respectively.

The focus here will be on the PPARα isoform. PPARα is a type-II non-steroid ligand-regulated nuclear hormone receptor [4,32,62] transcribed from the human PPARA gene, which spans ~93.2 kb [53] and consists of eight exons [63]. It has been mapped to chromosome 15 in the mouse DNA and to chromosome 22 in humans [31]. The PPARα protein possesses five main functional domains (A–F) embodied in a modular canonical structure [64] (Figure 4). The N-amino terminal end harbors the activation function-1 (AF-1) or A/B domain, which operates autonomously in a ligand-independent manner. The 65 amino acid-long DNA-binding domain (DBD), or C domain, consists of 2 highly conserved zinc finger-like motifs that promote the receptor’s binding to the PPRE sequence of the target genes. The D domain or hinge region that bridges the DBD to the ligand-binding domain (LBD) acts as a docking site for cofactors. In the C-terminal region, the LBD, or E/F domain, is responsible for ligand specificity and contains the activation function 2 (AF-2) [28,65]. This latter contains a tyrosine residue on the helix 12, which plays an ultimate role in interacting with the carboxyl group of the ligands [66]. When a ligand enters the LBD pocket of PPARα, the interface of AF-2 stabilizes and facilitates so that PPARα can recruit coactivators [67]. The LBD is still a center of interest in numerous pharmaceutical investigations. Recent publications to date on studies based on X-ray crystallography, referenced in the protein data bank website (PDB; http://www.pdb.org/, accessed on 1 July 2021), provide fascinating, detailed insight into the LBD domain structure, albeit limited to comparing it with other PPAR receptors. It describes a relatively large Y-shaped hydrophobic cavity in the PPARα-LBD pocket volume of 1400 Å^3^ [68], which allows PPARα to interact with a broad range of structurally distinct natural and synthetic ligands [67,69].

## 5. PPARα Ligands

Recently, PPARα-ligands have gained consistent interest in several complex metabolic disease investigations [60,67], such as lipid metabolism disorders. Due to their engagement in physiological and pathophysiological metabolic processes, and their role in activating transcriptional regulatory networks, these ligands are becoming intriguing bona fide treatment opportunities and present a way to unveil many relevant potential roles of PPARα, also known as promising versatile drug targets.

Evidence indicates that a wide variety of lipophilic molecules, the so-called ligands, can activate PPARα, encompassing natural saturated, unsaturated, and polyunsaturated fatty acids (PUFAs) [70,71], and synthetic ligands that are collectively referred to as PPARα-activators [72].

### 5.1. PPARα Natural Ligands

**Figure 5:**
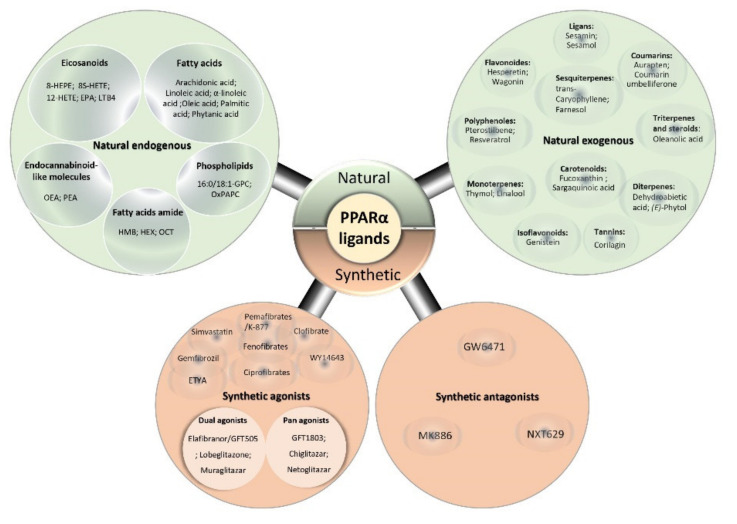
Diagram of different types and classes of PPARα ligands. The natural ligands type encompasses endogenous natural ligands (fatty acids [28,80,81,82], eicosanoids [31,83,84,85], phospholipids [75,86], fatty-acid amide [87] and endocannabinoid-like molecules [77], and exogenous natural ligands [30,88] (polyphenol flavonoids, isoflavonoids, monoterpenes, sesquiterpenes, diterpenes, triterpenes and steroids, carotenoids, coumarins, ligans, and tannins). The synthetic ligands type includes various classes of synthetic agonists [28,31,62,67,69,88,89,90,91] with various activation and binding modes (single [88,92], dual [28,91,93,94] and pan agonists [28,92]), and synthetic antagonists [89,90,95]. Abbreviations: 8-HEPE: 8-hydroxyeicosapentaenoic acid, 12-HETE: 12-hydroxyeicosatetraenoic acid, 8S-HETE: 8 (S)-hydroxyeicosatetraenoic acids, 16:0/18:1-GPC: phosphatidylcholine(1-palmitoyl-2-oleoyl-sn-glycerol-3-phosphocholine), EPA: eicosapentaenoic acid (20:5), ETYA: eicosatetraynoic acid, HEX: hexadecanamide, HMB: 3-hydroxy-(2,2)-dimethyl butyrate, OCT:9-octadecenamide, OEA: oleoyl-ethanolamide, OxPAPC: oxidized 1-palmitoyl-2-arachidonoyl-sn-glycero-3-phosphocholine, PEA: palmitoyl-ethanolamine.

Natural ligands include endogenous metabolites products derived from the lipid metabolism, such as acyl CoAs [73,74], oxidized fatty acids [63], phospholipids [75], certain nitrated derivatives of fatty acids, eicosanoids [76], endocannabinoid-like molecules [77], and lipoprotein lipolytic products [78]. PPARα natural activators could also originate from an exogenous source that is either found in dietary constituents [65], e.g., dietary ω-3 polyunsaturated fatty acids (docosahexaenoic acid and eicosapentaenoic acid) or issuing from traditionally used medicinal plants (reviewed by Rigano et al. [79]) (Figure 5).

Numerous findings provide evidence that natural ligands exhibit different binding affinities, which subsequently impact PPARα activation potency. Previous reports showed that omega-3 eicosapentaenoic acid (20:5, ω3) and, to a lesser extent, docosahexaenoic acid (22:6, ω3), are potent ligands [96,97] and consistent activators of PPARα [98,99,100], while omega-3 PUFA like linolenic acid (C18:3, ω3) and docosapentaenoic (22:5, ω3) acids, and omega-6 PUFA such as linoleic (C18:2, ω6) and arachidonic (C20:4, ω6) acids are weaker PPARα activators [74,99,100]. In addition, experiments performed by Ellinghaus et al. and Zomer et al. [101,102] revealed that phytanic acid (3,7,11,15-tertamethylhexadecanoic acid) is a strong natural physiological ligand for PPARα. These assumptions were then followed by reports from Hostetler et al. [103], showing that PPARα binds the fatty acyl-CoAs (3ߝ20 nM Kds) and branched-chain fatty acyl-CoA (BCFA-CoAs, phytanoyl-CoA, pristanoyl-CoA; Kds near 11 nM) with the highest affinities (i.e., Kd at nM range).

Natural PPARα ligands description studies, using full-length- or chimeric LBD- PPARα constructs, revealed the ability of many saturated and unsaturated fatty acids to activate target gene expression through PPARα modulation. Several PPARα-responsive genes are involved in fatty acid oxidation: (i) mitochondrial β-oxidation pathway (i.e., carnitine palmitoyltransferase 1A) [104]; (ii) microsomal ω-hydroxylation (i.e., CYP4A1-subclass of cytochrome P450 enzymes); and (iii) peroxisomal β-oxidation pathway (i.e., ACOX1; enoyl-CoA hydratase/3-hydroxyacyl-CoA dehydrogenase [105], 3-ketoacyl-CoA Thiolase and SCPx) [7,106,107].

The abovementioned results were recently supported by Chen et al. [108], reporting that feeding animals a diet high in rapeseed oil (rich in erucic acid, a very-long-chain fatty acid) leads to PPARα activation with an adaptive elevation in peroxisomal β-oxidation capacity, which suggested that erucic acid might act as a potential ligand for PPARα. In line with prior communicated data, Maheshwari et al. [109] reported that treating rat Fao cells with a fungal lipid extract rich in monomethyl BCFAs (*Conidiobolus heterosporous*) increases mRNA levels of the PPARα target genes *Acox1*, *Cyp4a1*, *Cpt1A*, and *Slc22A5*, strongly suggesting that BCFAs are similarly potent PPARα activators [109]. Taken together, these relevant results from our laboratory and from others all affirm that the peroxisomal β-oxidation substrates are potent PPARα ligands that modulate the expression of a battery of lipid-metabolizing enzymes to maintain lipid homeostasis and to alleviate the toxic effect of VLCFA and BCFA overload [57].

### 5.2. PPARα Synthetic Ligands

In the same way, PPARα binds to synthetic ligands termed PPARα activators. Interestingly, PPARα-activators exhibit structural features like a carboxylic acid head and a hydrophobic tail, connected via an aliphatic chain and a central aromatic ring [101]. This group of compounds includes various insecticides (2,4-dichlorophenoxyacetic acid); herbicides (phenoxyacetate derivatives) [110]; surfactants (perfluorooctanoic acid-PFOA); organic chlorinated hydrocarbons solvents such as perchloroethylene and trichloroethylene [111]; food flavors [112]; leukotriene D4 receptor antagonists [113]; phthalate plasticizers, such as di-(2-ethylhexyl)-phthalate and di-(2-ethylhexyl) adipate [114]; and amphipathic carboxylic acids [98]. The latter form the hypolipidemic fibrate class of drugs, acknowledged as the archetypal PPARα agonists, including clofibrates [88,89]; pemafibrates [67,69]; fenofibrates [67], and ciprofibrates [115]. It is notable that certain synthetic ligands are designed to act as dual agonists, like muraglitazar [93], that target both PPARα and PPARγ isotypes; others act as pan-agonists that activate all PPAR receptors like bezafibrates [92]; or as a PPARα partial agonist such as GW9662 [69], known as a PPARγ-selective antagonist (Figure 5). Interestingly, GW9662 displays dual effects by acting as agonist and antagonist against PPARα and also has the ability to enhance agonistic activities of certain less potent fibrates [69], whereas PPARα antagonists like GW6471 [89], MK886 [90], and NXT629 [95] represent the rare range of synthetic ligands that prevent other molecules from binding to this nuclear receptor.

To date, various synthetic single, dual and pan agonists, respectively, are in clinical use as medications to treat dyslipidemia, hyperglycemia in patients with Type 2 diabetes mellitus, hypertriglyceridemia, and cardiovascular disease [28,72,91]. Indeed, potent synthetic ligands could elicit both desirable and undesirable side effects. Studies conducted by Preiss et al. [116] proved that the chronic administration of peroxisome proliferators to rodents causes hepatocellular carcinoma, and it may also increase the risk of gallstones and cause anemia and leukopenia [117]. Much of what we know about PPARα-ligands comes from a collective knowledge primarily derived from rodent studies, via the treatment of mice or rats with synthetic PPARα peroxisome proliferators or by using PPARα null mice [98]. It has been reported that human and mouse PPARα have different binding affinities and physiological effects [118] and are diversely activated by specific ligands, including phthalates and fibrates [119]. Nevertheless, these differences are negligible and do not call into question the tenet of the ultimate role that PPARα plays as a general lipid sensor in both species [98].

To date, tremendous efforts are in progress to develop new, highly PPARα-specific ligands with different activation and binding modes that could more selectively activate PPARα–RXRα transcriptional complex assembly, with tissue-selective and gene-selective activities, to reduce unwanted side effects and assure reasonable safety. In parallel, the “micronutrients” found in food that activate PPAR receptors are gaining increasing interest, as nutritional therapy becomes an unstoppable trend for treating lipid disorders [79].

## 6. PPARα and Coregulators

**Figure 6:**
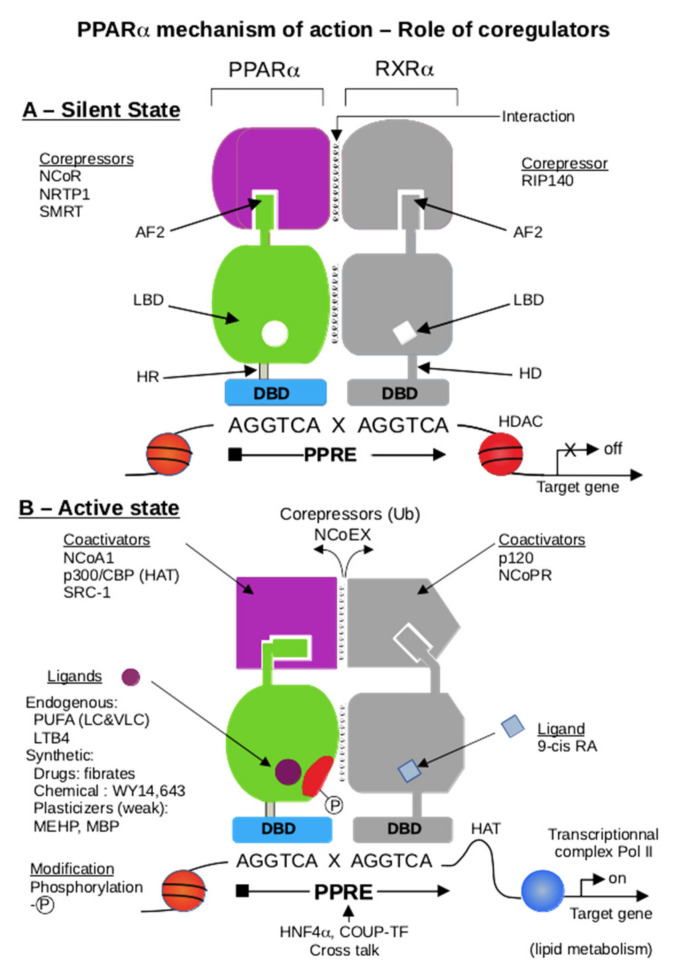
Scheme of heterodimer PPARα/RXRα, located on a PPRE DNA region. The graphic displays two parts: part (**A**), the silent state, and part (**B**), the active state. Part (**A**). In the absence of the ligand, PPARα interacts with transcriptional corepressors (NCoR, SMRT, NRTR-P-1) by recognizing the AF2 region. The same process is at the RXR, where AF2 interacts with RIP140 as RXRα corepressor [169]. Due to the chromatin condensed state, the heterodimer cannot bind the PPRE properly. Part (**B**). In the presence of a PPARα ligand, either a long-chain or very-long-chain polyunsaturated fatty acid, leukotriene LTB4, fibrate or other chemicals [170], and a 9-cis retinoic acid as RXRα ligand, an exchange corepressor/coactivator is made by NCoEX, which suppresses the repressive of corepressor state by ubiquitinylation-inducing degradation by the proteasome system. The fixation of a ligand induces an allosteric LBD conformational change of AF2, allowing the recruitment of coactivators, either NCoR1, p300/CBP, or SRC1 for PPARα, and p120 and NCoPR for RXRα [171]. The CBP-dependent HAT activity induces the remodeling of chromatin and allows the PPARα/RXRα heterodimer to bind to PPRE correctly, then activates the Pol II transcription complex and triggers the transcription of lipid metabolism-encoding genes. Some post-translational modifications of PPARα regulate its activity [172,173]. For instance, phosphorylation stimulates PPARα transcriptional activity [174]. The HNF4α transcription factor recognizes a similar response element as the PPRE and interplay with PPARα [175]. A comparable mechanism has been reported with the Coup-TF transcription factor. While several works consider PGC-1α [176] as an important coregulator of PPARα, it seems to be more specific for PPAR γ. The 15(S)-HETE, 15-hydroxyicosatetraenoic acid, family of arachidonic acid metabolites; 9-cisRA, retinoic acid cis conformation in carbon 9; AF1, activating domain 1; AF2, activating domain 2; CBP, CREBP binding protein; CoPRs, COPR1 and COPR2 as corepressors of PPAR and RXR, respectively; COUP-TF, chicken ovalbumin upstream promoter transcription factor; CTBP-2, C-terminal binding protein-2; DBD, DNA binding domain; HAT, histone acetyl-transferase; HD, hinge domain; HNF-4α, hepatic nuclear factor 4 α; HDAC, histone de-acetyl transferase; LBD, ligand binding domain; LTB4, leukotrien B4; MBP, mono butyl phthalate; MEHP, mono ethyl hexyl phthalate; NCoA1, nuclear receptor coactivator 1; NCoEX, nuclear receptor corepressor Excit; NCoR1, nuclear receptor corepressor 1; NRTP-1, nuclear repressor transcription factor; p120, protein 120 kDa; p300, protein 300 kDa; Pol II, RNA polymerase class II; PGC-1αPPAR γ co-activator-1α; PPRE, peroxisome proliferator response element; PRIP/RAP250, PPAR interacting-protein methyl transferase; PUFA (LC &VLC), polyunsaturated fatty acids (long-chain or very-long-chain); RIP140 receptor interacting protein corepressor; SMRT, silencing mediator of retinoid and thyroid receptors; SRC1, steroid receptor coactivator-1.

The identification of PPAR in the 1990s heralded a new era of biotic and xenobiotic sensing by the liver [4]. The PPAR subfamily of nuclear receptors functions as sensors for fatty acids and fatty acid derivatives and controls critical metabolic pathways involved in lipid and energy metabolism [120,121] and catabolism [122,123,124,125]. The transcriptional activation of genes is a complex process that involves the participation of many transcription factors [126]. While the nuclear receptors (NRs) mediated gene-regulation provide the backbone for the transcription factor-specific gene regulation, coregulators provide the much-needed tissue-, cell-, and species-specific differences in the peroxisome proliferator-induced pleiotropic responses of PPARα [127,128]. However, we would like to focus this review section on the role of PPARα and its associated proteins in regulating peroxisomal beta-oxidation genes/pathways. Coregulators are proteins that bind to the nuclear receptor by a specific domain LXXLL, a hallmark for all coregulators [129]. Most coregulators have more than one LXXLL domain and are essential for protein–protein interactions between the nuclear receptor and the coregulator [130] (Figure 6). Moreover, each LXXLL could function in a specific nuclear interaction, suggesting that the coregulators are shared between different NRs.

**Figure 7:**
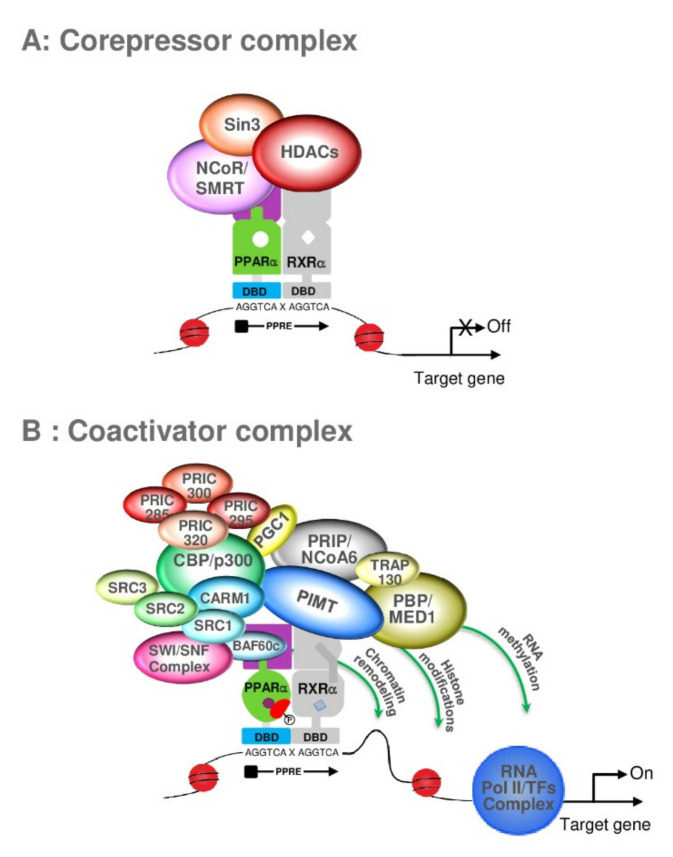
Interaction of PPARα-RXRα heterodimer with corepressor complex (**A**) or coactivator complex (**B**). (**A**) The corepressor complex, including Sin 3, NCoR/SMRT, and HDAC proteins, is recruited to an unliganded PPARα-RXRα heterodimer, so there is no transcription of the PPARα-target genes. (**B**) in the presence of PPARα-ligand, the PPARα-RXRα heterodimer exhibits a conformational change, leading to the dissociation of the corepressor complex, the recruitment of coactivator proteins, and the binding of PPARα to the peroxisome proliferator response element (PPRE). Different subcomplex modules participate in chromatin remodeling, through the acetylation (SRCs, p300) and the methylation (CARM1) of nucleosomes. Mediator components interact with PPARα and promote the recruitment of the basal transcription factors (TFs) to establish a connection with the RNA polymerase II to transcription of PPARα-target genes.

Coregulators can be broadly classified into subgroups, such as essential vs. non-essential, repressors vs. activators, and DNA binding region (DBD) vs. ligand binding-region (LBD) interacting coregulators [127]. Essential coregulators are proteins deemed critical for the survival of the offspring, and their absence results in embryonic lethality: cAMP-response element-binding protein (CBP); PPAR-interacting protein/activating signal cointegrator 2 (PRIP/ASC2); PPAR-binding protein/mediator complex subunit 1 (PBP/Med1); mediator complex subunit 25 (Med25) [131,132,133]. Non-essential coregulators are proteins with such critical functional responsibilities that they are usually represented by more than one isoform—steroid receptor coactivators (SRCs) [131,132,134], Asp-Glu-Ala-Asp (DEAD)-box helicases [135,136,137], sirtuins (SIRT) [96,97], PPARγ coactivators (PGCs) [138,139,140,141]—and the loss of one isoform is compensated by others. Repressors that bind to the nuclear receptor PPARα in the absence of/or independent of ligands prevent it from binding to the peroxisomal proliferator response elements (PPRE) of the target genes as nuclear corepressor (NCoR) and silencing mediator of retinoic acid and thyroid hormone (SMRT) [96] (Figure 7A). This group of proteins usually bind to the AF-1 domain of the DNA binding region of the receptor. The ligand-independent coregulators (heat-shock protein-70) could also prevent the PPARα from proteolytic degradation in the cytosol before PPARα could translocate to the nucleus in the activated state [131] and, typically, these proteins bind to the hinge region of the nuclear receptor that interconnects the DNA binding region to the ligand-binding region of the receptor [127]. The activators, on the other hand, could help PPARα zero in onto the specific PPREs of the target genomic region, help attach it to the PPREs with the assistance of nucleosomal-specific functions such as histone methylases (SRC proteins) [142], histone acetyltransferases (CBP/p300) [143,144], DNA-helicases [145], PRIC285 [124], and PRIC320 [146]. The activators would also function by stabilizing the transcriptional complex (PRIP/ASC2 [147]) and potentiate the recruitment of RNA-polymerase complex proteins to the transitional complex (mediator complex, PBP [148,149] (Figure 7B). Additionally, the activators would consist of proteins responsible for separating the transcribed mRNA from the genomic region (protein-L-isoaspartate (D-aspartate) O-methyltransferase (PIMT) [147]. These proteins activate the AF-2 domain of the nuclear receptor and enhance transcription by linking the liganded nuclear receptor to the basal transcription machinery. We have identified almost all these groups of coregulators using either a direct protein–protein interaction assay, such as a yeast two-hybrid assay [150], GST-pull downs [124], and ligand affinity chromatography [141] to identify the PPARα-interacting proteins and a functional transcriptional activation complex [131]. PPARs, like other nuclear receptors, interact with coactivators such as SRC-1 (steroid receptor coactivator-1) or corepressors such as NCoR and SMRT. PPARα-interacting coactivators and corepressors augment or repress, respectively, the PPARα transactivation activity. Since the cloning of SRC-1 twenty-five years ago, over 300 coactivators/coregulators have been identified, with new members still being added to this expanding spectrum. PPARα is known to interact with some of these coregulators [151]. These include CBP/p300-dependent binding complex [152], members of the SRC/p160 superfamily, members of PBP/MED1 complex (PBP/TRAP220/DRIP205/MED1 [133,149,153], members of PRIP/NCoA6 (ASC2/RAP250/TRBP/NRC), members of PRIC complex PRIC285 [124], PRIC295 [141], PRIC320 [146], PPAR gamma-binding proteins, PGC-1α [147,154], and PGC-1β [155,156], as well as coactivator-associated proteins PIMT [131] (NCoA6IP) and coactivator-associated arginine methyltransferase 1 (CARM-1) [157,158]. The PPARα-interacting coregulator (PRIC) complex isolated from rat liver nuclear extracts reveals many coregulators, presumably forming one mega-complex. An almost similar complex was isolated with ciprofibrate as the ligand in affinity chromatography. This diversity raises several issues about the evolutionary importance of the versatility and complexity of coregulatory molecules, their relative abundance in various cell types, and their affinity for a given nuclear receptor in orchestrating transcription in gene-, cell-, and developmental stage-specific transcription. In the absence of a specific ligand, PPARα interacts with the corepressors NCoR and SMRT, but the importance of PPARα action is not well documented, as endogenous ligands could potentially activate PPARα [159]. The homozygous deletion of NCoR or SMRT in mice is embryonically lethal, indicating that they cannot fully compensate for each other during development [160,161,162]. Furthermore, another corepressor, the receptor-interacting protein 140 (RIP140), which can interact with PPARα, is known to repress the activity of NRs by competing with coactivators and by recruiting downstream effectors such as histone deacetylases (HDACs) [163]. Interestingly, the phenotype of RIP140 knockout mice suggests a role for this corepressor in PPARα signaling, as these mice exhibit resistance to high-fat diet-induced obesity, resulting from the upregulation of genes involved in energy dissipation [163]. Interestingly, hepatic sirtuin 1 (SIRT1) regulates lipid homeostasis by positively regulating PPARα [164,165]. On the other hand, SIRT1 interacts with PPARγ and is regulated by PPARγ in a negative feedback mechanism [166]. SIRT6 binds NCOA2, a PPARα coactivator and part of the SRC family of coactivators; the binding results in the decrease of the acetylation of SRC2/NCOA2 K780 in the liver, thus, interaction with SIRT6 mediates the activation of PPARα and thus the inhibition of SREBP-dependent cholesterol and triglyceride synthesis [167]. The ligand binding to a nuclear receptor triggers a molecular switch that releases corepressors and begins the recruitment of coactivator complexes, such as members of the CBP/p300 family, which exhibit the histone acetyltransferase activity required to facilitate chromatin remodeling. The subsequent recruitment of other coregulators, either singly or as preassembled multi-subunit protein complexes, including mediator complex and RNA polymerase machinery, is facilitated by the interaction of the general basal transcription machinery to enhance the transcription of a specific set of genes [97,168]. As discussed previously, coregulators contain an LXXLL motif that forms two turns of the α-helix and binds to a hydrophobic cleft on the surface of the nuclear receptor. The identification and characterization of coregulators have been derived mostly from in vitro experiments, but there is a paucity of information about individual coactivators in vivo cell- and gene-specific functional roles [131].

## 7. Metabolic Regulation of the Peroxisomal β-Oxidation Pathways

The regulation of the peroxisomal pathways is mainly associated with the cellular increase of the peroxisome population, which is highly promoted by several diverse natural and synthetic compounds nominated as peroxisome proliferators (PPs). Such compounds raise a peroxisome number quantitatively, mainly in hepatic parenchymal cells, and provoke delayed pleiotropic responses, including the development of hepatocarcinoma in rats and mice [8,131]. Based on several pieces of experimental evidence, earlier reports from Reddy’s group proposed a receptor-mediated mechanism to explain the phenomenon of hepatic peroxisome proliferation. Accordingly, the induction of peroxisomal β-oxidation is a consequence of ligand hepatic overload, leading to lipid metabolism dysregulation, accompanied by an augmentation in the extrahepatic lipolysis and a substantial hepatic influx of free fatty acids [96]. Furthermore, the unique pleiotropic responses raised by structurally unrelated peroxisome proliferators in hepatocytes drive a synchronized transcriptional activation of the peroxisomal β-oxidation genes [13,96,131].

Lazarow and De Duve [7] demonstrated previously that clofibrate administration in rat liver strikingly enhances the peroxisomal β-oxidation activity. A similar observation was reported by Hashimoto and coworkers [177], showing that feeding a diet containing a phthalate ester plasticizer di-(2-ethylhexyl)phthalate, a PPARα activator, leads to a 20-fold increase in the expression of peroxisomal β-oxidation enzymes in rat liver. In addition, a previous study reported that synthetic ligands such as WY-14643 exhibited a high affinity to PPARα, compared to the natural endogenous ligand (16:0/18:1-GPC) in the induction of fatty acid β-oxidation [75]. Moreover, Rogue et al. [93] showed that *Acox1* and *Cpt1A* genes in oleic-acid-overloaded HepaRG cells were significantly upregulated from 1 day, and remained at high levels after 14 days, upon treatment with the dual agonist muraglitazar, which stimulates the fatty acid β-oxidation pathway. These results are in close concordance with previous experiments conducted by Lee et al. [126], showing that after feeding hypolipidemic agents to mice lacking PPARα expression, the mutant animals accumulated lipid droplets in their tissues, which strongly supports the idea that PPARα activators promote the transcription of genes involved in the lipid catabolism process.

Structurally, PPs molecules may be chemically unrelated, including hypolipidemic drugs, such as clofibrate, ciprofibrate, gemfibrozil, and Wy-14,643, as well as some nutritional conditions, especially high-fat diet or vitamin E-deficient diet and leukotriene D4 receptor antagonists. In addition, several herbicides, such as 2,4-dicholophenoxyacetic acid or 4-chloro-2-methylphenoxyacetic acid [8,178] and certain phthalate ester plasticizers, induce a similar liver peroxisome proliferation as do prototypic fibrate derivatives. In addition, the administration to rodents of a C19-steroid, dehydroepiendrosterone, promotes peroxisomal fatty acid β-oxidation and peroxisome proliferation [179]. Though the response to PPs has been demonstrated in several tissues from PPs-treated rodents, the hepatic responsiveness is by far the most powerful, accounting for a 10- to 20-fold induction of peroxisomal fatty acid β-oxidation activities, accompanied by a proliferation of peroxisomes and strong hepatomegaly pathogenesis [8,131].

**Figure 8:**
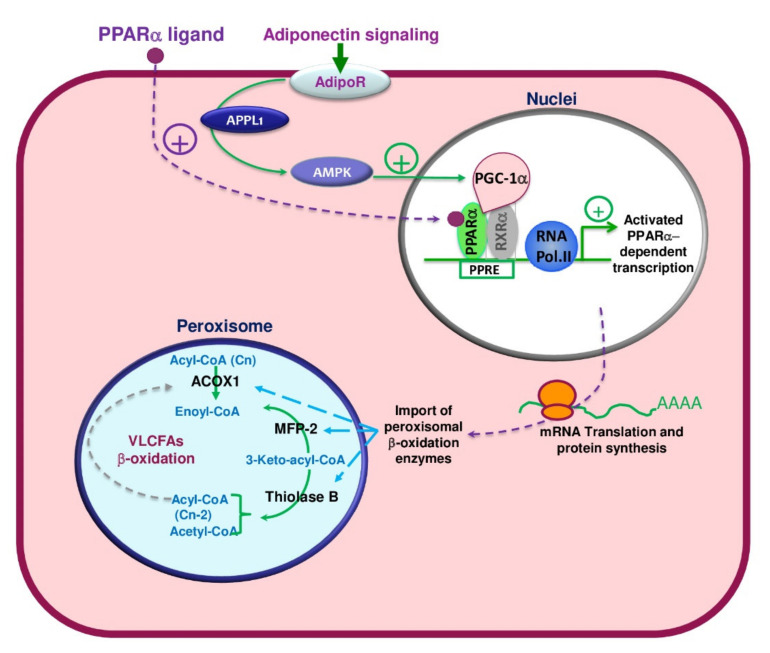
PPARα-dependent regulation of peroxisomal fatty acid b-oxidation in rat liver through adiponectin signaling. Adiponectin: a hormone produced by adipose tissue that plays a role in lipid and glucose metabolism regulation; AdipoR, adiponectin receptor; APPL1, an adaptor protein containing a PH domain, PTB domain, and leucine zipper motif 1, plays a central role as the main contributing factor in the adiponectin and insulin signaling; AMPK, AMP kinase; PGC1- α, PPAR γ coactivator1α; PPRE, peroxisome proliferator-activated receptor; RNA Pol II, RNA-polymerase II; ACOX1, acyl-CoA oxidase 1; MFP2, multifunctional protein 2; thiolase B, 3-ketoacyl-CoA thiolase B; VLCFA, very-long-chain fatty acid.

The description of PPARα-target genes shows that this nuclear hormone receptor largely governs those genes involved in hepatic and cardiac muscle transport, oxidation, and the degradation of lipids. Transcriptionally, PPARα activates several genes, including the lipoprotein lipase gene permitting the release of fatty acids from lipoprotein particles [180], genes encoding fatty acid translocase CD36, and fatty acid-binding protein-facilitating fatty acids capture and transport them through the plasma membrane [8,180]. The acyl-CoA synthetase, activating fatty acids to acyl-CoAs, is another gene-target of PPARα [96,98]. Regarding the genes encoding peroxisomal β-oxidation enzymes, the induction of the peroxisomal fatty acyl-CoA ABC transporter D2 (ALDRP) by PPs was shown to be partially PPARα-dependent in mice hepatocytes [179]; however, the regulation of, e.g., ACOX1, L-PBE and ThB, are entirely reliant on PPARα [8,98,181]. Nevertheless, the regulation of genes implicated in the mitochondrial fatty acid β- oxidation, including the carnitine palmitoyltransferase-1 and the medium chain-acyl-CoA dehydrogenase, is also coordinated by PPARα [98,182,183]. Thus, PPARα arises as a master regulator controlling the hepatic metabolism of free fatty acids. The development of PPARα null mice evidenced the crucial role played by PPARα in the concerted regulation of peroxisome proliferation and expression of its target genes involved in both β- and ω-fatty acid oxidations [181]. By contrast to *Ppara*^-/-^ mice, which exhibit mild hepatic steatosis, *Acox1* null mice develop strong hepatic steatosis, showing a hepatic peroxisome proliferation and the sustained activation of PPARα and expression of its target genes [147,184]. Thus, paradoxically, the defect in ACOX1 activity leads to the hepatic accumulation of ACOX1 substrates, of which some have been shown [147] as efficient endogenous PPARα ligands, mediating the sustained activation of PPARα. On the other hand, the strong PPARα activation of fatty acid β-oxidation genes increases hepatic dicarboxylic acid production and accumulation. Thus, in the absence of ACOX1 activity, these dicarboxylic acids are still unmetabolized and act as firm inhibitors of mitochondrial fatty acid β-oxidation [185]. Moreover, the *Ppara*^-/-^, *Acox1*^-/-^ double-knockout mice exhibit a few periportal clusters of steatotic hepatocytes, and (re-)expression of human *ACOX1* in mice liver results in a substantial reduction in both PPARα activation and hepatic steatosis [8,180]. Peroxisomal fatty acid β-oxidation is induced by starvation in a PPARα-dependent manner, as validated by its impairment in PPARα null mice [8,180]. Accordingly, the deacetylase sirtuin-1 is dispensable to PPARα-inducing peroxisomal fatty acid β-oxidation and needs SIRT1-PPARα interaction, and the deletion of hepatic SIRT1 negatively impacts PPARα signaling [165]. The MAP kinase kinase kinase TGFβ-activated kinase 1 (TAK1) acts upstream to PPARα, and its deletion also impaired the PPARα-dependent induction of peroxisomal fatty acid β-oxidation [186]. PPARα signaling has also been shown to involve the AMPK-SIRT1-PGC-1α axis via the adiponectin receptors [187] (Figure 8). These results strongly highlight the detrimental role of the peroxisomal β-oxidation pathway in the sensing of PPARα activity.

Several peroxisomal β-oxidation substrates display a substantial role as PPARα modulators. It is believed that the activities of (inducible and non-inducible) peroxisomal fatty acid β-oxidation systems are modulated by PPARα [108]. Moreover, several findings provide significant evidence that VLCFA and BCFA, which are considered potentially toxic fatty acids, are potent inducers of PPARα that enhance the transcription of peroxisomal enzymes mediating fatty acid β-oxidation [57,188].

Interestingly, Oleoylethanolamide, a naturally occurring lipid regulating satiety and body weight, exhibited a high-affinity binding to PPARα and the activation of its lipid-metabolizing target genes [189]. Nonetheless, we should consider that most fatty acids are subject to elongation, desaturation, esterification, and β-oxidation, which could modify the availability of PPARα ligands. Accordingly, very-long-chain saturated and unsaturated fatty acids are exclusively metabolized by peroxisomal β-oxidation, which participates in their degradation, synthesis, or retro conversion. One defect in this pathway is associated with the accumulation of VLCFAs and a deficit in certain PUFAs’ synthesis, such as DHA. Interestingly, a mouse deficiency of ACOX1, the rate-limiting enzyme in the peroxisomal β-oxidation, leads to the sustained activation of hepatic PPARα and the induction of its target genes [190]. The role of ACOX1 in PPARα lipid sensing was highlighted by *Acox1^-/-^; ob/ob* double knockout mice. Thus, the sustained activation of PPARα when linked to the absence of ACOX1 activity attenuates the metabolic consequences of leptin deficiency, due to the *ob/ob* genotype, showing less obesity with the recovery of glucose homeostasis and alleviating insulin resistance [131,147]. Collectively, accumulated data underline the key role of peroxisomal β-oxidation in sensing PPARα-dependent lipid and energy metabolism.

## 8. PPARα Expression in Species and Tissue Distribution

### 8.1. PPARα Expression in Different Species

PPAR is ubiquitous among animal species, i.e., worms [191], insects, fish, frogs [192], reptiles, mammals, including hamsters [193], and humans. A PPARab subtype was detected in zebrafish. This PPARab mutant shows lower expression in liver and visceral mass, which were associated with lipid accumulation [194]. In a jerboa (*Jaculus orientalis*) liver, both active wild-type PPARα (PPARα1 wt) and a truncated PPARα 2 forms were expressed. The availability of active PPARα1 wt is differentially regulated during fasting-associated hibernation [195].

### 8.2. PPARα Tissue Distribution

PPARα tissue expression is also ubiquitous, although on a different level. PPARα is mainly expressed in tissues with high rates of fatty acid catabolism, i.e., those involved in digestive function (liver, stomach, enterocytes) and muscular activity (heart, skeletal muscle, kidney at proximal tubules). In the nervous system, the expression is moderated (low in retinal, or lacking expression in the central nervous system). Low expression is found in the pancreas and adipose tissue [196], while in the brain, PPARα is found at the highest levels in neurons, followed by astrocytes, and is weakly expressed in microglia [62,197]—more likely, to upregulate the expression of several synaptic related genes coding proteins engaged in excitatory neurotransmission and the neuroprotective mechanism [198,199,200]. In the immune system, PPARα expression is detected in the spleen, monocytes/macrophages, and neutrophils [201]. In addition, expression is seen in reproductive organs and the epidermis. PPARα is also associated with tumorigenesis in colorectal carcinoma [202]. Concerning the expression in developmental tissue in rats, *Ppara* transcripts are detectable in mouse embryo at 13.5 gestation days, to reach the maximum level at birth [203].

### 8.3. Lessons from Pparα Knockout

This part of the manuscript provides recent findings from the last five years related to *Ppara* knockout animals, with the intent of disentangling the PPARα’s various biological functions in health and disease and to evaluate its engagement in fatty acid catabolism and clearance in liver and heart tissues, where PPARα and FAO are both abundant. A growing body of evidence indicates that PPARα is a crucial regulator of systemic lipid metabolism. PPARα deficiency is considered to be a prime factor that either causes or exacerbates fatty acid metabolism impairment, which leads inevitably to the development of numerous metabolic diseases, to name but a few—obesity [204,205], type 2 diabetes mellitus, insulin resistance, dyslipidemia, myocardial infarction, hepatic steatosis without ethanol consumption, termed non-alcoholic fatty liver disease (NAFLD), which includes severe phenotypes such as non-alcoholic steatohepatitis (NASH), liver fibrosis, and hepatocellular carcinoma [206,207,208,209,210]. Therefore, many investigations were conducted using mainly PPARα knockout mouse models, because of the relative equivalent expression of *Ppara* mRNA between mice and humans in different tissues [98]. Knockout animal models are generated either with the global (*Ppara*^-/-^) or hepatocyte-specific abrogation of the *Ppara* gene, such as *Ppara^Hep-/-^* (reviewed by Wang et al. [181]). The goal was to identify the pathophysiological mechanisms underlying the abnormal phenotypes associated with PPARα dysfunction and to assess the distinct contribution of hepatic and extrahepatic PPARα to global energy and immune system homeostasis in vivo.

### 8.4. Lessons from Pparα-KO in the Liver

Hepatic PPARα activation occurs during suckling [211], with a high-fat diet, and during fasting [212,213,214], boosting fatty acid oxidation (FAO), which participates in the restoration of energy homeostasis and provides energy supply for the extrahepatic tissues. For that reason, most of the studies were focused on hepatic PPARα. Furthermore, hepatic PPARα can protect the liver against fasting/high-fat diet-induced steatosis, by transactivating the genes required for fatty acid catabolism and repressing several inflammatory genes. Thus, during the fasting process, metabolic substrates stored in white adipose tissue are released into the circulation and captured by the liver. Subsequently, this increases β-oxidation and ketogenesis to maintain the energy balance [212]. It was observed that fasted *Ppara^-/-^* and *Ppara^Hep-/-^* mice developed hypoketonemia, hypoglycemia, and hypothermia with decreased serum triglycerides. Additionally, the ectopic accumulation of medium-chain fatty acids and long-chain fatty acids in the liver manifests as an increase of hepatic fat mass, termed steatosis, with pronounced oxidative stress and lipid peroxidation compared to wild-type mouse liver. These effects result from the altered mitochondrial and peroxisomal fatty acid β-oxidation pathways in the liver [212,214,215]. Furthermore, mice in which *Ppara* was deleted uniquely in hepatocytes could not modulate bone marrow monocyte egress upon fasting [216], suggesting that PPARα contributes to the regulation of monocyte homeostasis during fasting.

Regarding high-fat diet (HFD)-induced obesity, mice with the hepatocyte-specific deletion of *Ppara* develop steatosis and inflammation [217]. These observations corroborate previous results communicated by Stec and al. [205], showing that *Ppara^Hep-/-^* mice on HFD had worsened hepatic inflammation associated with steatosis, and exhibited high levels of LDL, which is considered an emerging risk factor for cardiovascular disease in NAFLD. PPARα could also protect against obesity. In *ob/ob* obese mice, the absence of PPARα resulted in increased obesity and led to severe hepatic steatosis [184]. Interestingly, mice lacking only hepatocyte-PPARα developed steatosis spontaneously but without obesity in aging [212,214]. Indeed, extrahepatic PPARα activity blunts and compensates when hepatic PPARα is disrupted, by elevating FAO and lipase activity in other tissues to increase and utilize excess lipid, thus maintaining lipid homeostasis [215]. Likewise, the transcriptome, lipidome, and metabolome results communicated by Régnier et al. and Batatinha et al. [217,218] demonstrate the significant contribution of extrahepatic PPARα activity to the metabolic homeostasis response to HFD consumption. By using double-knockout mice, *Ppara^-/-^/Cyp2a5^-/-^*, Chen et al. [108,206] together indicate that PPARα interacts with CYP2a5 (cytochrome P450 2A5) an antioxidant enzyme to protect against steatosis. Fibroblast growth factor 21 (FGF21) acts as a downstream molecule of the PPARα signaling pathway to regulate the liver lipid metabolism and contribute to the CYP2a5 protective effects on alcoholic fatty liver disease [206]. In an experiment conducted by Brocker et al. [219], it was observed that treatment with WY-14643, a PPARα agonist, caused weight loss and severe hepatomegaly in WT and *Ppara^ΔMac^* mice but not in *Ppara^Hep-/-^* and *Ppara^-/-^* mice, suggesting that cell proliferation is mediated exclusively by PPARα activation in hepatocytes in response to WY-14643 agonist treatment.

*Pparab* is one of the two *Ppara* paralogs, highly expressed in zebrafish tissues with high oxidative activity. Li and coworkers generated *Pparab-knockout* in the zebrafish model [194]. *Pparab*-null zebrafish demonstrated a lower expression of critical enzymes involved in FAO, and lower mitochondrial and peroxisomal FAO in the liver and muscle, associated with lipid accumulation in the liver. Furthermore, PPARab deficiency increases glucose oxidation, protein synthesis, and reduced amino acid breakdown, while in rodents, the loss of PPARα increases amino acid breakdown [194].

### 8.5. Lessons from Ppara-KO in the Heart

PPARα is a crucial regulator of substrate utilization in the heart. Fatty acids are a primary energy source for the heart, and fatty acid β-oxidation provides almost 70% of cardiac ATP; the remainder is obtained primarily from glycolysis and lactate oxidation [220]. Thus, *Ppara* KO mice, in response to chronic pressure overload, exhibit enhanced cardiac dysfunction. In contrast, mild PPARα activation in mice showed a positive effect on myocardial energetic functions, especially during progressive and pressure-overloaded heart failure, revealing the virtue of PPARα-associated FAO modulation as a promising therapeutic strategy for heart failure [221]. In addition, *Ppara* ablation exacerbated myocardial ischemia-reperfusion injury in *Ppara* KO mice models subjected to cardiac ischemia-reperfusion, and interestingly, after the treatment with PEA microparticles (PEA-um^®®^, 10 mg/Kg), an endogenous PPARα ligand, only *Ppara* WT mice showed the cardioprotective effect of PEA-um^®®^, but not in *Ppara* KO mice. Although PEA-um^®®^ had a protective and beneficial effect on inflammatory disorders associated with ischemic myocardial failure, it also negatively regulates inflammation through PPARα activation by reducing the activation of the nuclear factor-kB (NF-kB) pathway and production of pro-inflammatory cytokines [222]. Thus, PPARα could augment heart function and cardiac fatty acid oxidation, whereas in the *Ppara* KO mouse model, a more severe sepsis phenotype is observed due to deteriorated cardiac performance and fatty acid oxidation, associated with both a hyperinflammatory cytokine storm as well as immune paralysis [223]. Furthermore, during sepsis, WT hearts showed a decrease in PPARα and other FAO genes’ mRNA expression, and this reduction was more dramatic in *Ppara*-*null* mouse hearts [223]. Taken together, PPARα expression increased fatty acid oxidation and subsequently supported the hyperdynamic cardiac response early during sepsis or pressure-overloaded heart failure, which may prevent morbidity and mortality.

## 9. PPARα and Micronutrients

As reported above, PPARα is activated by different ligands of both natural and synthetic origins, involved in several signaling and metabolic pathways. Some natural ligands are issued from the lipid metabolism, such as PUFAs and their derivatives. Interestingly, micronutrients, such as minerals, vitamins, phytophenols, and phytosterols are non-energetic compounds with essential signaling activity. Of particular interest, polyphenols, oil products, and some terpenoids and alkaloids impact cell functions through the modulation of PPARα activity.

## 10. Effect of Polyphenols, Known as Antioxidants and Anti-Aging Compounds

### 10.1. Resveratrol

Resveratrol, or 3,4′,5-trihydroxystilbene, is a natural polyphenol present in large amounts in Japanese knotweed (*Polygonum cuspidatum*) root. This phytoalexin is produced by a wide variety of plants, some of which are edible for humans, such as grapes, blackberries, blackcurrants, blueberries, and cranberries, to name but a few [224]. However, in the last two decades, the effect of resveratrol on animal models related to several disorders, such as autism spectrum disorder, mitochondrial myopathies, type 2 diabetic nephropathy, or renal lipotoxicity has been increasingly reported.

The effect of resveratrol in the presence of quercetin has been studied on PPARα-mediating uncoupling protein regulation in visceral white adipose tissue from metabolic syndrome rats. Resveratrol treatment leads to a significantly increased expression of both *Ppara* mRNA and protein levels [225]. Remarkably, resveratrol prevents renal lipotoxicity in a high-fat diet-treated mouse model by regulating the PPARα pathway, enhancing the expression of lipolytic genes, and raising the renal PPARα protein level and AMPK phosphorylation level [226]. Due to known dyslipidemia in autism spectrum disorders, PPARs have been proposed as therapeutic targets of resveratrol. Furthermore, in autism, impaired mitochondrial fatty acid oxidation suggests the potential implications for regulating mitochondrial oxidation flux by PPAR activators, especially resveratrol [227].

Numerous natural ligands, including polyphenolic compounds, control the expression of PPAR receptors [228]. They have several health-promoting properties, including antioxidant, anti-inflammatory, and antineoplastic activities. Resveratrol is an active biological modulator of several signaling proteins, including PPARα. Resveratrol activates the AMPK-SIRT1-PGC-1α axis and PPARα via the adiponectin receptors in the renal cortex [187]. Adiponectin has multiple functions, including insulin sensitization and lipid metabolism regulation. Similarly, in mitochondrial myopathy, resveratrol has been shown to potentially target many mitochondrial metabolic pathways comprising fatty acid β-oxidation and oxidative phosphorylation, leading to the up-regulation of the energy supply via AMPkinase-SIRT1-PGC-1α signaling pathways [229].

### 10.2. Quercetin

Quercetin (2-(3,4-dihydroxyphenyl)-3,5,7-trihydroxy-4H-chromen-4-one) is a flavonoid polyphenol found in plants and a variety of other natural sources—red grape, onion, broccoli, tomatoes and lettuce [224]. PPARα is significantly upregulated and enhances β-oxidation by mulberry-leaf powder containing quercetin [230]. Quercetin-3-*O*-β-d-glucuronide (Q3GA) ameliorates dyslipidemia in fatty livers by modulating the PPARα/sterol regulatory element-binding protein-1c (SREBP-1c) signaling. Q3GA reduced lipogenesis through downregulation of SREBP-1c and fatty acid synthase levels, and raised lipolysis and fatty acid oxidation by increasing the expression of PPARα, carnitine palmitoyl-transferase1 and medium-chain acyl-coenzyme A dehydrogenase, both in vivo and in vitro [231].

### 10.3. EGCG (Epigallocatechin-3-Gallate)

Epigallocatechin-3-gallate (EGCG) is catechin conjugated with gallic acid. It belongs to the flavonol class and is found abundantly in green tea [232] and cocoa, which have the highest content of catechins, followed by prune juice, broad bean pods, and argan oil [224].

EGCG and green tea polyphenol extract display crosstalk with PPARα. Reported studies in cancer cells revealed that EGCG induced the expression level of PPARα protein in a dose-dependent manner. Clofibrate, a PPARα agonist, blocks heme oxygenase-1 (HO-1) induction and sensitizes cancer cells to EGCG-promoted cell death. Moreover, PPARα interacts with the PPRE of the HO-1 promoter. The activation of PPARα sensitizes cancer cells to epigallocatechin-3-gallate (EGCG) treatment by suppressing HO-1 expression [233]. In rats, green tea polyphenols reduce the renal oxidative stress induced by a high-fat diet through deacetylation of SIRT3 mediated by PPARα upregulation [234].

### 10.4. Curcumin

Curcumin (1,7-bis-(4-hydroxy-3-methoxyphenyl)-hepta-1,6-diene-3,5-dione) belongs to a chemical class of polyphenols that is extracted from the rhizomes of the turmeric plant (*Curcuma longa*) [224]. Tetrahydro-curcumin improves oleic acid-induced hepatic steatosis and ameliorates insulin resistance in HepG2 cells, likely through downregulation of the expression of the lipogenic proteins, SREBP-1c and PPARγ, and the stimulation of lipolysis by upregulating PPARα and CPT-1a, which are involved in fatty acid β-oxidation [235].

### 10.5. Anthocyanins

Among berries, blueberries contain higher amounts of anthocyanins. These polyphenols are known to exhibit hypolipidemic properties. Rimando et al. reported that both anthocyanins and catechins do not activate PPARα, while pterostilbene revealed the dose-dependent activation of PPARα in H4IIEC3 hepatocytes [236]. In addition, pterostilbene showed a significant increase in *Ppara* gene expression, but at a lower extent than fenofibrate [236]. Although pterostilbene and resveratrol, as PPARα activators, are under the threshold for effective concentrations in blueberry extract, hepatic mRNA *Ppara* expression has increased in hamsters fed on a diet containing blueberry extract [236].

### 10.6. Coffee

Coffee consumption has been shown to upregulate mouse hepatic PPARα expression and its target-gene *Acox1*, consequently leading to the induction of liver peroxisomal fatty acid β-oxidation. Such FAO induction, with induced intestinal cholesterol efflux and reduced lipid digestion, prevents the high-fat diet-induced fatty liver through the lipid-sensing modulation of the gut–liver axis [237].

### 10.7. Edible Oil Products

The effect of polyphenols has been investigated in a rat model of bowel disease by 3 months diet supplementation with extra-virgin olive oil with a high or low phenolic content [238]. The presence of polyphenols in olive oil significantly attenuates the intestinal inflammation associated with hypocholesterolemia and the induction of PPAR-α gene expression in the liver [238]. In a model of insulin resistance of rats fed a high-fat diet, the administration of the major metabolite of oleuropein, hydroxytyrosol, increases the hepatic mRNA levels of *Ppara* and its target genes, i.e., fibroblast growth factor 21 and carnitine palmitoyltransferase 1a [239]. Similarly, mice receiving a high-fat diet develop hepatic steatosis and inflammation, which were attenuated by hydroxytyrosol supplementation through PPARα activation, Nrf2 (nuclear factor, erythroid 2 like 2) mediated-antioxidative pathway, and by the downregulation of NF-κB-associated inflammation [240]. Used as food supplementation, argan oil or olive oil was shown to restore the expression of genes involved in liver mitochondrial and peroxisomal fatty acid β-oxidation and gluconeogenesis in the mice sepsis model when injected with lipopolysaccharides. This preventive effect of argan oil likely involves the hepatic upregulation of PPARα, PGC-1α, and the estrogen-related receptor α [241].

Likewise, ginsenoside Rb3 micronutrients, derived from ginseng, or nuciferine, found in *Nelumbo nucifera* leaves, was shown to activate the PPARα pathway by regulating energy metabolism in cardiomyocytes [242], or hepatic steatosis diabetic streptozocin-induced mice fed a high-fat diet [243], while bilobetin, a biflavonoid, modulates PPARα activity by PKA-dependent phosphorylation. Finally, berberine, an alkaloid, binds PPARα LBD with a hypolipidemic effect and a comparable affinity to fenofibrate [244].

## 11. Conclusions and Future Directions

In all these tested situations, irrespective of the tissue, animal, or pathological condition, micronutrients appear to have an advantageous effect on *Ppara* expression and activity. Furthermore, almost all these compounds are potent antioxidants and can activate signaling pathways via PGC1-α and AMP kinase. Numerous natural products might modulate PPARα, including terpenes, polyketides, phenylpropanoids, polyphenols, and alkaloids; for instance, the linalool effect is ten times less compared to fenofibrate [88], demonstrating the potential beneficial effects of dietary micro-components to modulate PPARα functions desirably in a population with an ever-increasing high-fat diet consumption. The question is the dietary relevance of these effects, since most of the data were obtained from in vitro studies, and secondly, these micronutrients are often present at very low doses in the diet, except for some polyphenols.

Despite tremendous signs of progress on the critical role of PPARα-dependent regulation in lipid metabolism, the characterization of peroxisomal enzymes and transporters, there are still gaps that need to be filled to fully define the exact role and regulation of PPARα and peroxisomal fatty acid β-oxidation in the cellular homeostasis of lipid metabolism. Particular attention needs to be focused on:

1. The shuttling of substrates and cofactors from and into peroxisome.
2. What is the exact role of peroxisomal β-oxidation in lipid metabolism and cell signaling?
3. How can peroxisome be a mediator and responder of metabolic and environmental stresses?
4. What are the molecular events that are required at the metabolic level?
  (a) Does heterodimerization of PPAR/RXR control the regulation? Is it controlled by coregulators?
  (b) What is the nature of ligands?
  (c) What is the nature of micronutrients? Are they natural agonists or antagonists or their balance?
  (d) Is PPARα the only nuclear receptor governing peroxisomal β-oxidation-related genes?
  (e) How do coregulators play in concert to fine-tune metabolically peroxisomal β-oxidation pathway?

All these as yet unanswered questions deserve our complete focus in the near future.

There is an increasing demand from health institutions and pharmaceutical industries for efficient drugs. PPARα binding pocket-ligand interactions are being increasingly recognized as a source for therapeutic interventions. Bio structural analysis based on X-ray crystallography and ligand structure pharmacophore modeling approaches afford new biophysical and structural parameters that are important in designing and developing novel potent and highly PPARα-specific ligands to preserve human health and safety. However, the overall goal of increasing the peroxisomal fatty acid oxidation and β-oxidation safely, without increasing the lipid peroxidation and free radical-based risk of non-genotoxic carcinogenesis in the high-fat Western diet-fed population, is a challenge that is still unmet and requires continuous exploration of avenues to activate PPARα dependent pathways safely.

## Figures and Tables

**Table 1:** Peroxisomal genes experimentally proved to be regulated through the PPARα binding to PPREs.

Gene	Protein	Species	PPRE ^a^	PPRE Sequence ^b^	Reference
*Acaaa1b*	acetyl-coenzyme A acyltransferase 1B	rat	PPRE2	AGGTCA **A** AAGTCA	[40]
*ACOX1*	acyl-CoA oxidase 1	human	PPRE	AGGTCA **G** CTGTCA	[41]
*Acox1*	acyl-CoA oxidase 1	rat	PPRE	TGACCT **T** TGTCCT	[36]
*Aldh3a2*	aldehyde dehydrogenase family 3, subfamily A2	mouse	PPRE	nd	[42]
*Cat*	catalase	rat	PPRE	AGGTGA **A** AGTTGA	[43]
*Ehhadh*	enoyl-CoA hydratase and 3-hydroxyacyl CoA dehydrogenase	rat	PPRE	TGACCT **A** TTGAAC	[44]
*Mlycd*	malonyl-CoA decarboxylase	rat	PPRE-2	AGGCAA **G** AGGCTG	[45]
*Mlycd*	malonyl-CoA decarboxylase	rat	PPRE-3	GAACCT **T** TGGCTG	[45]
*Pex11a*	peroxisomal biogenesis factor 11 alpha	mouse	PPRE	TCACCT **T** TCACCC	[46]
*Scp2*	sterol carrier protein 2	rat	PPRE-A	TCCTGT **A** ACTCCG	[47]
*Scp2*	sterol carrier protein 2	rat	PPRE-B	GTGGAT **T** ACAGGA	[47]

^a^ corresponds, for each gene, to the PPRE numbering as stated in the corresponding reference. ^b^ PPREs sequence: PPARα DR-1 sequences are shown with hexads underlined and spacing nucleotides in bold.

## Data Availability

Not applicable.

## References

[1] Latruffe N., Vamecq J. (2000). Evolutionary Aspects of Peroxisomes as Cell Organelles, and of Genes Encoding Peroxisomal Proteins. Biol. Cell.

[2] Hess R., Stäubli W., Riess W. (1965). Nature of the Hepatomegalic Effect Produced by Ethyl-Chlorophenoxy-Isobutyrate in the Rat. Nature.

[3] Lalwani N.D., Reddy M.K., Qureshi S.A., Sirtori C.R., Abiko Y., Reddy J.K. (1983). Evaluation of Selected Hypolipidemic Agents for the Induction of Peroxisomal Enzymes and Peroxisome Proliferation in the Rat Liver. Hum. Toxicol..

[4] Issemann I., Green S. (1990). Activation of a Member of the Steroid Hormone Receptor Superfamily by Peroxisome Proliferators. Nature.

[5] Zhou T., Yan X., Wang G., Liu H., Gan X., Zhang T., Wang J., Li L. (2015). Evolutionary Pattern and Regulation Analysis to Support Why Diversity Functions Existed within PPAR Gene Family Members. BioMed Res. Int..

[6] Wanders R.J., Waterham H.R., Ferdinandusse S. (2015). Metabolic Interplay between Peroxisomes and Other Subcellular Organelles Including Mitochondria and the Endoplasmic Reticulum. Front. Cell Dev. Biol..

[7] Lazarow P.B., De Duve C. (1976). A Fatty Acyl-CoA Oxidizing System in Rat Liver Peroxisomes; Enhancement by Clofibrate, a Hypolipidemic Drug. Proc. Natl. Acad. Sci. USA.

[8] Cherkaoui-Malki M., Surapureddi S., El-Hajj H.I., Vamecq J., Andreoletti P. (2012). Hepatic Steatosis and Peroxisomal Fatty Acid Beta-Oxidation. Curr. Drug Metab..

[9] Andreoletti P., Raas Q., Gondcaille C., Cherkaoui-Malki M., Trompier D., Savary S. (2017). Predictive Structure and Topology of Peroxisomal ATP-Binding Cassette (ABC) Transporters. Int. J. Mol. Sci..

[10] Watkins P.A., Ellis J.M. (2012). Peroxisomal Acyl-CoA Synthetases. Biochim. Biophys. Acta.

[11] Caira F., Clémencet M.C., Cherkaoui-Malki M., Dieuaide-Noubhani M., Pacot C., Van Veldhoven P.P., Latruffe N. (1998). Differential Regulation by a Peroxisome Proliferator of the Different Multifunctional Proteins in Guinea Pig: CDNA Cloning of the Guinea Pig D-Specific Multifunctional Protein 2. Biochem. J..

[12] Osumi T., Ishii N., Hijikata M., Kamijo K., Ozasa H., Furuta S., Miyazawa S., Kondo K., Inoue K., Kagamiyama H. (1985). Molecular Cloning and Nucleotide Sequence of the CDNA for Rat Peroxisomal Enoyl-CoA: Hydratase-3-Hydroxyacyl-CoA Dehydrogenase Bifunctional Enzyme. J. Biol. Chem..

[13] Latruffe N. (2020). Human Peroxisomal 3-Ketoacyl-CoA Thiolase: Tissue Expression and Metabolic Regulation: Human Peroxisomal Thiolase. Adv. Exp. Med. Biol..

[14] Baes M., Van Veldhoven P.P. (2016). Hepatic Dysfunction in Peroxisomal Disorders. Biochim. Biophys. Acta.

[15] Van Veldhoven P.P., De Schryver E., Young S.G., Zwijsen A., Fransen M., Espeel M., Baes M., Van Ael E. (2020). Slc25a17 Gene Trapped Mice: PMP34 Plays a Role in the Peroxisomal Degradation of Phytanic and Pristanic Acid. Front. Cell Dev. Biol..

[16] Seedorf U., Brysch P., Engel T., Schrage K., Assmann G. (1994). Sterol Carrier Protein X Is Peroxisomal 3-Oxoacyl Coenzyme A Thiolase with Intrinsic Sterol Carrier and Lipid Transfer Activity. J. Biol. Chem..

[17] Ranea-Robles P., Violante S., Argmann C., Dodatko T., Bhattacharya D., Chen H., Yu C., Friedman S.L., Puchowicz M., Houten S.M. (2021). Murine Deficiency of Peroxisomal L-Bifunctional Protein (EHHADH) Causes Medium-Chain 3-Hydroxydicarboxylic Aciduria and Perturbs Hepatic Cholesterol Homeostasis. Cell. Mol. Life Sci..

[18] Wang H., Lu J., Chen X., Schwalbe M., Gorka J.E., Mandel J.A., Wang J., Goetzman E.S., Ranganathan S., Dobrowolski S.F. (2021). Acquired Deficiency of Peroxisomal Dicarboxylic Acid Catabolism Is a Metabolic Vulnerability in Hepatoblastoma. J. Biol. Chem..

[19] Tillander V., Alexson S.E.H., Cohen D.E. (2017). Deactivating Fatty Acids: Acyl-CoA Thioesterase-Mediated Control of Lipid Metabolism. Trends Endocrinol. Metab..

[20] Bowen K.J., Kris-Etherton P.M., Shearer G.C., West S.G., Reddivari L., Jones P.J.H. (2017). Oleic Acid-Derived Oleoylethanolamide: A Nutritional Science Perspective. Prog. Lipid Res..

[21] Desvergne B., Michalik L., Wahli W. (2006). Transcriptional Regulation of Metabolism. Physiol. Rev..

[22] Varga T., Czimmerer Z., Nagy L. (2011). PPARs Are a Unique Set of Fatty Acid Regulated Transcription Factors Controlling Both Lipid Metabolism and Inflammation. Biochim. Biophys. Acta.

[23] Mandard S., Müller M., Kersten S. (2004). Peroxisome Proliferator-Activated Receptor Alpha Target Genes. Cell. Mol. Life Sci..

[24] More V.R., Campos C.R., Evans R.A., Oliver K.D., Chan G.N., Miller D.S., Cannon R.E. (2017). PPAR-α, a Lipid-Sensing Transcription Factor, Regulates Blood-Brain Barrier Efflux Transporter Expression. J. Cereb. Blood Flow Metab..

[25] Ning L.-J., He A.-Y., Lu D.-L., Li J.-M., Qiao F., Li D.-L., Zhang M.-L., Chen L.-Q., Du Z.-Y. (2017). Nutritional Background Changes the Hypolipidemic Effects of Fenofibrate in Nile Tilapia (*Oreochromis Niloticus*). Sci. Rep..

[26] Martin G., Schoonjans K., Lefebvre A.M., Staels B., Auwerx J. (1997). Coordinate Regulation of the Expression of the Fatty Acid Transport Protein and Acyl-CoA Synthetase Genes by PPARalpha and PPARgamma Activators. J. Biol. Chem..

[27] Motojima K., Passilly P., Peters J.M., Gonzalez F.J., Latruffe N. (1998). Expression of Putative Fatty Acid Transporter Genes Are Regulated by Peroxisome Proliferator-Activated Receptor Alpha and Gamma Activators in a Tissue- and Inducer-Specific Manner. J. Biol. Chem..

[28] Han L., Shen W.-J., Bittner S., Kraemer F.B., Azhar S. (2017). PPARs: Regulators of Metabolism and as Therapeutic Targets in Cardiovascular Disease. Part I: PPAR-α. Future Cardiol..

[29] Kandel B.A., Thomas M., Winter S., Damm G., Seehofer D., Burk O., Schwab M., Zanger U.M. (2016). Genomewide Comparison of the Inducible Transcriptomes of Nuclear Receptors CAR, PXR and PPARα in Primary Human Hepatocytes. Biochim. Biophys. Acta.

[30] Lefebvre P., Benomar Y., Staels B. (2010). Retinoid X Receptors: Common Heterodimerization Partners with Distinct Functions. Trends Endocrinol. Metab..

[31] Wójtowicz S., Strosznajder A.K., Jeżyna M., Strosznajder J.B. (2020). The Novel Role of PPAR Alpha in the Brain: Promising Target in Therapy of Alzheimer’s Disease and Other Neurodegenerative Disorders. Neurochem. Res..

[32] Corrales P., Vidal-Puig A., Medina-Gómez G. (2018). PPARs and Metabolic Disorders Associated with Challenged Adipose Tissue Plasticity. Int. J. Mol. Sci..

[33] Dreyer C., Krey G., Keller H., Givel F., Helftenbein G., Wahli W. (1992). Control of the Peroxisomal Beta-Oxidation Pathway by a Novel Family of Nuclear Hormone Receptors. Cell.

[34] Feige J.N., Gelman L., Tudor C., Engelborghs Y., Wahli W., Desvergne B. (2005). Fluorescence Imaging Reveals the Nuclear Behavior of Peroxisome Proliferator-Activated Receptor/Retinoid X Receptor Heterodimers in the Absence and Presence of Ligand. J. Biol. Chem..

[35] Kliewer S.A., Umesono K., Noonan D.J., Heyman R.A., Evans R.M. (1992). Convergence of 9-Cis Retinoic Acid and Peroxisome Proliferator Signalling Pathways through Heterodimer Formation of Their Receptors. Nature.

[36] Tugwood J.D., Issemann I., Anderson R.G., Bundell K.R., McPheat W.L., Green S. (1992). The Mouse Peroxisome Proliferator Activated Receptor Recognizes a Response Element in the 5′ Flanking Sequence of the Rat Acyl CoA Oxidase Gene. EMBO J..

[37] Tzeng J., Byun J., Park J.Y., Yamamoto T., Schesing K., Tian B., Sadoshima J., Oka S. (2015). An Ideal PPAR Response Element Bound to and Activated by PPARα. PLoS ONE.

[38] Fornes O., Castro-Mondragon J.A., Khan A., Van Der Lee R., Zhang X., Richmond P.A., Modi B.P., Correard S., Gheorghe M., Baranašić D. (2020). JASPAR 2020: Update of the Open-Access Database of Transcription Factor Binding Profiles. Nucleic Acids Res..

[39] Koch J., Pranjic K., Huber A., Ellinger A., Hartig A., Kragler F., Brocard C. (2010). PEX11 Family Members Are Membrane Elongation Factors That Coordinate Peroxisome Proliferation and Maintenance. J. Cell Sci..

[40] Hansmannel F., Clémencet M.-C., Le Jossic-Corcos C., Osumi T., Latruffe N., Nicolas-Francés V. (2003). Functional Characterization of a Peroxisome Proliferator Response-Element Located in the Intron 3 of Rat Peroxisomal Thiolase B Gene. Biochem. Biophys. Res. Commun..

[41] Woodyatt N.J., Lambe K.G., Myers K.A., Tugwood J.D., Roberts R.A. (1999). The Peroxisome Proliferator (PP) Response Element Upstream of the Human Acyl CoA Oxidase Gene Is Inactive among a Sample Human Population: Significance for Species Differences in Response to PPs. Carcinogenesis.

[42] Ashibe B., Motojima K. (2009). Fatty Aldehyde Dehydrogenase Is Up-Regulated by Polyunsaturated Fatty Acid via Peroxisome Proliferator-Activated Receptor Alpha and Suppresses Polyunsaturated Fatty Acid-Induced Endoplasmic Reticulum Stress. FEBS J..

[43] Girnun G.D., Domann F.E., Moore S.A., Robbins M.E.C. (2002). Identification of a Functional Peroxisome Proliferator-Activated Receptor Response Element in the Rat Catalase Promoter. Mol. Endocrinol..

[44] Bardot O., Aldridge T.C., Latruffe N., Green S. (1993). PPAR-RXR Heterodimer Activates a Peroxisome Proliferator Response Element Upstream of the Bifunctional Enzyme Gene. Biochem. Biophys. Res. Commun..

[45] Lee G.Y., Kim N.H., Zhao Z.-S., Cha B.S., Kim Y.S. (2004). Peroxisomal-Proliferator-Activated Receptor Alpha Activates Transcription of the Rat Hepatic Malonyl-CoA Decarboxylase Gene: A Key Regulation of Malonyl-CoA Level. Biochem. J..

[46] Shimizu M., Yamashita D., Yamaguchi T., Hirose F., Osumi T. (2006). Aspects of the Regulatory Mechanisms of PPAR Functions: Analysis of a Bidirectional Response Element and Regulation by Sumoylation. Mol. Cell. Biochem..

[47] Lopez D., Irby R.B., McLean M.P. (2003). Peroxisome Proliferator-Activated Receptor Alpha Induces Rat Sterol Carrier Protein x Promoter Activity through Two Peroxisome Proliferator-Response Elements. Mol. Cell. Endocrinol..

[48] Evans R.M., Barish G.D., Wang Y.-X. (2004). PPARs and the Complex Journey to Obesity. Nat. Med..

[49] Fan W., Evans R. (2015). PPARs and ERRs: Molecular Mediators of Mitochondrial Metabolism. Curr. Opin. Cell Biol..

[50] Green S., Wahli W. (1994). Peroxisome Proliferator-Activated Receptors: Finding the Orphan a Home. Mol. Cell. Endocrinol..

[51] Zhu Y., Qi C., Korenberg J.R., Chen X.N., Noya D., Rao M.S., Reddy J.K. (1995). Structural Organization of Mouse Peroxisome Proliferator-Activated Receptor Gamma (MPPAR Gamma) Gene: Alternative Promoter Use and Different Splicing Yield Two MPPAR Gamma Isoforms. Proc. Natl. Acad. Sci. USA.

[52] Göttlicher M., Widmark E., Li Q., Gustafsson J.A. (1992). Fatty Acids Activate a Chimera of the Clofibric Acid-Activated Receptor and the Glucocorticoid Receptor. Proc. Natl. Acad. Sci. USA.

[53] Sher T., Yi H.F., McBride O.W., Gonzalez F.J. (1993). CDNA Cloning, Chromosomal Mapping, and Functional Characterization of the Human Peroxisome Proliferator Activated Receptor. Biochemistry.

[54] Vamecq J., Latruffe N. (1999). Medical Significance of Peroxisome Proliferator-Activated Receptors. Lancet.

[55] Brown J.D., Plutzky J. (2007). Peroxisome Proliferator-Activated Receptors as Transcriptional Nodal Points and Therapeutic Targets. Circulation.

[56] Hong F., Pan S., Guo Y., Xu P., Zhai Y. (2019). PPARs as Nuclear Receptors for Nutrient and Energy Metabolism. Molecules.

[57] Lamichane S., Dahal Lamichane B., Kwon S.-M. (2018). Pivotal Roles of Peroxisome Proliferator-Activated Receptors (PPARs) and Their Signal Cascade for Cellular and Whole-Body Energy Homeostasis. Int. J. Mol. Sci..

[58] Moore J.T., Collins J.L., Pearce K.H. (2006). The Nuclear Receptor Superfamily and Drug Discovery. ChemMedChem.

[59] Floyd Z.E., Stephens J.M. (2012). Controlling a Master Switch of Adipocyte Development and Insulin Sensitivity: Covalent Modifications of PPARγ. Biochim. Biophys. Acta.

[60] Wadosky K.M., Willis M.S. (2012). The Story so Far: Post-Translational Regulation of Peroxisome Proliferator-Activated Receptors by Ubiquitination and SUMOylation. Am. J. Physiol. Heart Circ. Physiol..

[61] Kim T.-H., Kim M.-Y., Jo S.-H., Park J.-M., Ahn Y.-H. (2013). Modulation of the Transcriptional Activity of Peroxisome Proliferator-Activated Receptor Gamma by Protein-Protein Interactions and Post-Translational Modifications. Yonsei Med. J..

[62] Tufano M., Pinna G. (2020). Is There a Future for PPARs in the Treatment of Neuropsychiatric Disorders?. Molecules.

[63] Bougarne N., Weyers B., Desmet S.J., Deckers J., Ray D.W., Staels B., De Bosscher K. (2018). Molecular Actions of PPARα in Lipid Metabolism and Inflammation. Endocr. Rev..

[64] Pawlak M., Lefebvre P., Staels B. (2012). General Molecular Biology and Architecture of Nuclear Receptors. Curr. Top. Med. Chem..

[65] Lamas Bervejillo M., Ferreira A.M. (2019). Understanding Peroxisome Proliferator-Activated Receptors: From the Structure to the Regulatory Actions on Metabolism. Adv. Exp. Med. Biol..

[66] Oyama T., Toyota K., Waku T., Hirakawa Y., Nagasawa N., Kasuga J.I., Hashimoto Y., Miyachi H., Morikawa K. (2009). Adaptability and Selectivity of Human Peroxisome Proliferator-Activated Receptor (PPAR) Pan Agonists Revealed from Crystal Structures. Acta Crystallogr. D Biol. Crystallogr..

[67] Kawasaki M., Kambe A., Yamamoto Y., Arulmozhiraja S., Ito S., Nakagawa Y., Tokiwa H., Nakano S., Shimano H. (2020). Elucidation of Molecular Mechanism of a Selective PPARα Modulator, Pemafibrate, through Combinational Approaches of X-Ray Crystallography, Thermodynamic Analysis, and First-Principle Calculations. Int. J. Mol. Sci..

[68] Xu H.E., Lambert M.H., Montana V.G., Plunket K.D., Moore L.B., Collins J.L., Oplinger J.A., Kliewer S.A., Gampe R.T., McKee D.D. (2001). Structural Determinants of Ligand Binding Selectivity between the Peroxisome Proliferator-Activated Receptors. Proc. Natl. Acad. Sci. USA.

[69] Kamata S., Oyama T., Saito K., Honda A., Yamamoto Y., Suda K., Ishikawa R., Itoh T., Watanabe Y., Shibata T. (2020). PPARα Ligand-Binding Domain Structures with Endogenous Fatty Acids and Fibrates. iScience.

[70] Forman B.M., Chen J., Evans R.M. (1997). Hypolipidemic Drugs, Polyunsaturated Fatty Acids, and Eicosanoids Are Ligands for Peroxisome Proliferator-Activated Receptors Alpha and Delta. Proc. Natl. Acad. Sci. USA.

[71] Kliewer S.A., Sundseth S.S., Jones S.A., Brown P.J., Wisely G.B., Koble C.S., Devchand P., Wahli W., Willson T.M., Lenhard J.M. (1997). Fatty Acids and Eicosanoids Regulate Gene Expression through Direct Interactions with Peroxisome Proliferator-Activated Receptors Alpha and Gamma. Proc. Natl. Acad. Sci. USA.

[72] Takada I., Makishima M. (2020). Peroxisome Proliferator-Activated Receptor Agonists and Antagonists: A Patent Review (2014-Present). Expert Opin. Ther. Pat..

[73] Elholm M., Dam I., Jorgensen C., Krogsdam A.M., Holst D., Kratchmarova I., Gottlicher M., Gustafsson J.A., Berge R., Flatmark T. (2001). Acyl-CoA Esters Antagonize the Effects of Ligands on Peroxisome Proliferator-Activated Receptor Alpha Conformation, DNA Binding, and Interaction with Co-Factors. J. Biol. Chem..

[74] Hostetler H.A., Kier A.B., Schroeder F. (2006). Very-Long-Chain and Branched-Chain Fatty Acyl-CoAs Are High Affinity Ligands for the Peroxisome Proliferator-Activated Receptor Alpha (PPARalpha). Biochemistry.

[75] Chakravarthy M.V., Lodhi I.J., Yin L., Malapaka R.R.V., Xu H.E., Turk J., Semenkovich C.F. (2009). Identification of a Physiologically Relevant Endogenous Ligand for PPARalpha in Liver. Cell.

[76] Brown J.D., Karimian Azari E., Ayala J.E. (2017). Oleoylethanolamide: A Fat Ally in the Fight against Obesity. Physiol. Behav..

[77] Campolongo P., Roozendaal B., Trezza V., Cuomo V., Astarita G., Fu J., McGaugh J.L., Piomelli D. (2009). Fat-Induced Satiety Factor Oleoylethanolamide Enhances Memory Consolidation. Proc. Natl. Acad. Sci. USA.

[78] Azhar S. (2010). Peroxisome Proliferator-Activated Receptors, Metabolic Syndrome and Cardiovascular Disease. Future Cardiol..

[79] Rigano D., Sirignano C., Taglialatela-Scafati O. (2017). The Potential of Natural Products for Targeting PPARα. Acta Pharm. Sin. B.

[80] Green S. (1995). PPAR: A Mediator of Peroxisome Proliferator Action. Mutat. Res..

[81] Yu K., Bayona W., Kallen C.B., Harding H.P., Ravera C.P., McMahon G., Brown M., Lazar M.A. (1995). Differential Activation of Peroxisome Proliferator-Activated Receptors by Eicosanoids. J. Biol. Chem..

[82] Goto T., Takahashi N., Kato S., Egawa K., Ebisu S., Moriyama T., Fushiki T., Kawada T. (2005). Phytol Directly Activates Peroxisome Proliferator-Activated Receptor Alpha (PPARalpha) and Regulates Gene Expression Involved in Lipid Metabolism in PPARalpha-Expressing HepG2 Hepatocytes. Biochem. Biophys. Res. Commun..

[83] Wahli W., Michalik L. (2012). PPARs at the Crossroads of Lipid Signaling and Inflammation. Trends Endocrinol. Metab..

[84] Narala V.R., Adapala R.K., Suresh M.V., Brock T.G., Peters-Golden M., Reddy R.C. (2010). Leukotriene B4 Is a Physiologically Relevant Endogenous Peroxisome Proliferator-Activated Receptor-Alpha Agonist. J. Biol. Chem..

[85] Lin Q., Ruuska S.E., Shaw N.S., Dong D., Noy N. (1999). Ligand Selectivity of the Peroxisome Proliferator-Activated Receptor Alpha. Biochemistry.

[86] Delerive P., Furman C., Teissier E., Fruchart J., Duriez P., Staels B. (2000). Oxidized Phospholipids Activate PPARalpha in a Phospholipase A2-Dependent Manner. FEBS Lett..

[87] Roy A., Kundu M., Jana M., Mishra R.K., Yung Y., Luan C.-H., Gonzalez F.J., Pahan K. (2016). Identification and Characterization of PPARα Ligands in the Hippocampus. Nat. Chem. Biol..

[88] Bernardes A., Souza P.C.T., Muniz J.R.C., Ricci C.G., Ayers S.D., Parekh N.M., Godoy A.S., Trivella D.B.B., Reinach P., Webb P. (2013). Molecular Mechanism of Peroxisome Proliferator-Activated Receptor α Activation by WY14643: A New Mode of Ligand Recognition and Receptor Stabilization. J. Mol. Biol..

[89] Huang H.-T., Liao C.-K., Chiu W.-T., Tzeng S.-F. (2017). Ligands of Peroxisome Proliferator-Activated Receptor-Alpha Promote Glutamate Transporter-1 Endocytosis in Astrocytes. Int. J. Biochem. Cell Biol..

[90] Moraes L.A., Piqueras L., Bishop-Bailey D. (2006). Peroxisome Proliferator-Activated Receptors and Inflammation. Pharmacol. Ther..

[91] Mirza A.Z., Althagafi I.I., Shamshad H. (2019). Role of PPAR Receptor in Different Diseases and Their Ligands: Physiological Importance and Clinical Implications. Eur. J. Med. Chem..

[92] Tenenbaum A., Motro M., Fisman E.Z. (2005). Dual and Pan-Peroxisome Proliferator-Activated Receptors (PPAR) Co-Agonism: The Bezafibrate Lessons. Cardiovasc. Diabetol..

[93] Rogue A., Anthérieu S., Vluggens A., Umbdenstock T., Claude N., de la Moureyre-Spire C., Weaver R.J., Guillouzo A. (2014). PPAR Agonists Reduce Steatosis in Oleic Acid-Overloaded HepaRG Cells. Toxicol. Appl. Pharmacol..

[94] Shin N.-R., Park S.-H., Ko J.-W., Cho Y.-K., Lee I.-C., Kim J.-C., Shin I.-S., Kim J.-S. (2018). Lobeglitazone Attenuates Airway Inflammation and Mucus Hypersecretion in a Murine Model of Ovalbumin-Induced Asthma. Front. Pharmacol..

[95] Stebbins K.J., Broadhead A.R., Cabrera G., Correa L.D., Messmer D., Bundey R., Baccei C., Bravo Y., Chen A., Stock N.S. (2017). In Vitro and in Vivo Pharmacology of NXT629, a Novel and Selective PPARα Antagonist. Eur. J. Pharmacol..

[96] Duszka K., Gregor A., Guillou H., König J., Wahli W. (2020). Peroxisome Proliferator-Activated Receptors and Caloric Restriction-Common Pathways Affecting Metabolism, Health, and Longevity. Cells.

[97] Kosgei V.J., Coelho D., Gueant-Rodriguez R.M., Gueant J.L. (2020). Sirt1-PPARS Cross-Talk in Complex Metabolic Diseases and Inherited Disorders of the One Carbon Metabolism. Cells.

[98] Kersten S., Stienstra R. (2017). The Role and Regulation of the Peroxisome Proliferator Activated Receptor Alpha in Human Liver. Biochimie.

[99] Laleh P., Yaser K., Alireza O. (2019). Oleoylethanolamide: A Novel Pharmaceutical Agent in the Management of Obesity-an Updated Review. J. Cell. Physiol..

[100] Pawar A., Jump D.B. (2003). Unsaturated Fatty Acid Regulation of Peroxisome Proliferator-Activated Receptor Alpha Activity in Rat Primary Hepatocytes. J. Biol. Chem..

[101] Ellinghaus P., Wolfrum C., Assmann G., Spener F., Seedorf U. (1999). Phytanic Acid Activates the Peroxisome Proliferator-Activated Receptor Alpha (PPARalpha) in Sterol Carrier Protein 2-/Sterol Carrier Protein x-Deficient Mice. J. Biol. Chem..

[102] Zomer A.W., van Der Burg B., Jansen G.A., Wanders R.J., Poll-The B.T., van Der Saag P.T. (2000). Pristanic Acid and Phytanic Acid: Naturally Occurring Ligands for the Nuclear Receptor Peroxisome Proliferator-Activated Receptor Alpha. J. Lipid Res..

[103] Hostetler H.A., Petrescu A.D., Kier A.B., Schroeder F. (2005). Peroxisome Proliferator-Activated Receptor Alpha Interacts with High Affinity and Is Conformationally Responsive to Endogenous Ligands. J. Biol. Chem..

[104] Brady P.S., Marine K.A., Brady L.J., Ramsay R.R. (1989). Co-Ordinate Induction of Hepatic Mitochondrial and Peroxisomal Carnitine Acyltransferase Synthesis by Diet and Drugs. Biochem. J..

[105] Marcus S.L., Miyata K.S., Zhang B., Subramani S., Rachubinski R.A., Capone J.P. (1993). Diverse Peroxisome Proliferator-Activated Receptors Bind to the Peroxisome Proliferator-Responsive Elements of the Rat Hydratase/Dehydrogenase and Fatty Acyl-CoA Oxidase Genes but Differentially Induce Expression. Proc. Natl. Acad. Sci. USA.

[106] Reddy J.K., Mannaerts G.P. (1994). Peroxisomal Lipid Metabolism. Annu. Rev. Nutr..

[107] Zhang B., Marcus S.L., Miyata K.S., Subramani S., Capone J.P., Rachubinski R.A. (1993). Characterization of Protein-DNA Interactions within the Peroxisome Proliferator-Responsive Element of the Rat Hydratase-Dehydrogenase Gene. J. Biol. Chem..

[108] Chen X., Shang L., Deng S., Li P., Chen K., Gao T., Zhang X., Chen Z., Zeng J. (2020). Peroxisomal Oxidation of Erucic Acid Suppresses Mitochondrial Fatty Acid Oxidation by Stimulating Malonyl-CoA Formation in the Rat Liver. J. Biol. Chem..

[109] Maheshwari G., Ringseis R., Wen G., Gessner D.K., Rost J., Fraatz M.A., Zorn H., Eder K. (2020). Branched-Chain Fatty Acids as Mediators of the Activation of Hepatic Peroxisome Proliferator-Activated Receptor Alpha by a Fungal Lipid Extract. Biomolecules.

[110] Latruffe N., Cherkaoui Malki M., Nicolas-Frances V., Clemencet M.C., Jannin B., Berlot J.P. (2000). Regulation of the Peroxisomal Beta-Oxidation-Dependent Pathway by Peroxisome Proliferator-Activated Receptor Alpha and Kinases. Biochem. Pharmacol..

[111] Klaunig J.E., Babich M.A., Baetcke K.P., Cook J.C., Corton J.C., David R.M., DeLuca J.G., Lai D.Y., McKee R.H., Peters J.M. (2003). PPARalpha Agonist-Induced Rodent Tumors: Modes of Action and Human Relevance. Crit. Rev. Toxicol..

[112] Reddy J.K., Lalwai N.D. (1983). Carcinogenesis by Hepatic Peroxisome Proliferators: Evaluation of the Risk of Hypolipidemic Drugs and Industrial Plasticizers to Humans. Crit. Rev. Toxicol..

[113] Gonzalez F.J., Peters J.M., Cattley R.C. (1998). Mechanism of Action of the Nongenotoxic Peroxisome Proliferators: Role of the Peroxisome Proliferator-Activator Receptor Alpha. J. Natl. Cancer Inst..

[114] Maloney E.K., Waxman D.J. (1999). Trans-Activation of PPARalpha and PPARgamma by Structurally Diverse Environmental Chemicals. Toxicol. Appl. Pharmacol..

[115] Akbiyik F., Cinar K., Demirpence E., Ozsullu T., Tunca R., Haziroglu R., Yurdaydin C., Uzunalimoglu O., Bozkaya H. (2004). Ligand-Induced Expression of Peroxisome Proliferator-Activated Receptor Alpha and Activation of Fatty Acid Oxidation Enzymes in Fatty Liver. Eur. J. Clin. Investig..

[116] Preiss D., Tikkanen M.J., Welsh P., Ford I., Lovato L.C., Elam M.B., LaRosa J.C., DeMicco D.A., Colhoun H.M., Goldenberg I. (2012). Lipid-Modifying Therapies and Risk of Pancreatitis: A Meta-Analysis. JAMA.

[117] Estrela G.R., Arruda A.C., Torquato H.F.V., Freitas-Lima L.C., Perilhão M.S., Wasinski F., Budu A., Fock R.A., Paredes-Gamero E.J., Araujo R.C. (2020). Gemfibrozil Induces Anemia, Leukopenia and Reduces Hematopoietic Stem Cells via PPAR-α in Mice. Int. J. Mol. Sci..

[118] Oswal D.P., Balanarasimha M., Loyer J.K., Bedi S., Soman F.L., Rider S.D., Hostetler H.A. (2013). Divergence between Human and Murine Peroxisome Proliferator-Activated Receptor Alpha Ligand Specificities. J. Lipid Res..

[119] Oswal D.P., Alter G.M., Rider S.D., Hostetler H.A. (2014). A Single Amino Acid Change Humanizes Long-Chain Fatty Acid Binding and Activation of Mouse Peroxisome Proliferator-Activated Receptor α. J. Mol. Graph. Model..

[120] Chawla A., Repa J.J., Evans R.M., Mangelsdorf D.J. (2001). Nuclear Receptors and Lipid Physiology: Opening the X-Files. Science.

[121] Krey G., Braissant O., L’Horset F., Kalkhoven E., Perroud M., Parker M.G., Wahli W. (1997). Fatty Acids, Eicosanoids, and Hypolipidemic Agents Identified as Ligands of Peroxisome Proliferator-Activated Receptors by Coactivator-Dependent Receptor Ligand Assay. Mol. Endocrinol..

[122] Cave M.C., Clair H.B., Hardesty J.E., Falkner K.C., Feng W., Clark B.J., Sidey J., Shi H., Aqel B.A., McClain C.J. (2016). Nuclear Receptors and Nonalcoholic Fatty Liver Disease. Biochim. Biophys. Acta.

[123] Francque S., Szabo G., Abdelmalek M.F., Byrne C.D., Cusi K., Dufour J.F., Roden M., Sacks F., Tacke F. (2021). Nonalcoholic Steatohepatitis: The Role of Peroxisome Proliferator-Activated Receptors. Nat. Rev. Gastroenterol. Hepatol..

[124] Sinha R.A., Rajak S., Singh B.K., Yen P.M. (2020). Hepatic Lipid Catabolism via PPARalpha-Lysosomal Crosstalk. Int. J. Mol. Sci..

[125] Wagner N., Wagner K.-D. (2020). The Role of PPARs in Disease. Cells.

[126] Lee S.S., Pineau T., Drago J., Lee E.J., Owens J.W., Kroetz D.L., Fernandez-Salguero P.M., Westphal H., Gonzalez F.J. (1995). Targeted Disruption of the Alpha Isoform of the Peroxisome Proliferator-Activated Receptor Gene in Mice Results in Abolishment of the Pleiotropic Effects of Peroxisome Proliferators. Mol. Cell. Biol..

[127] Amber-Vitos O., Chaturvedi N., Nachliel E., Gutman M., Tsfadia Y. (2016). The Effect of Regulating Molecules on the Structure of the PPAR-RXR Complex. Biochim. Biophys. Acta.

[128] Surapureddi S., Yu S., Bu H., Hashimoto T., Yeldandi A.V., Kashireddy P., Cherkaoui-Malki M., Qi C., Zhu Y.J., Rao M.S. (2002). Identification of a Transcriptionally Active Peroxisome Proliferator-Activated Receptor Alpha -Interacting Cofactor Complex in Rat Liver and Characterization of PRIC285 as a Coactivator. Proc. Natl. Acad. Sci. USA.

[129] Skowron K.J., Booker K., Cheng C., Creed S., David B.P., Lazzara P.R., Lian A., Siddiqui Z., Speltz T.E., Moore T.W. (2019). Steroid Receptor/Coactivator Binding Inhibitors: An Update. Mol. Cell. Endocrinol..

[130] Surapureddi S., Rana R., Reddy J.K., Goldstein J.A. (2008). Nuclear Receptor Coactivator 6 Mediates the Synergistic Activation of Human Cytochrome P-450 2C9 by the Constitutive Androstane Receptor and Hepatic Nuclear Factor-4alpha. Mol. Pharmacol..

[131] Misra P., Reddy J.K. (2014). Peroxisome Proliferator-Activated Receptor-α Activation and Excess Energy Burning in Hepatocarcinogenesis. Biochimie.

[132] Rana R., Surapureddi S., Kam W., Ferguson S., Goldstein J.A. (2011). Med25 Is Required for RNA Polymerase II Recruitment to Specific Promoters, Thus Regulating Xenobiotic and Lipid Metabolism in Human Liver. Mol. Cell. Biol..

[133] Spitler K.M., Ponce J.M., Oudit G.Y., Hall D.D., Grueter C.E. (2017). Cardiac Med1 Deletion Promotes Early Lethality, Cardiac Remodeling, and Transcriptional Reprogramming. Am. J. Physiol. Heart Circ. Physiol..

[134] De Vera I.M.S., Zheng J., Novick S., Shang J., Hughes T.S., Brust R., Munoz-Tello P., Gardner W.J., Marciano D.P., Kong X. (2017). Synergistic Regulation of Coregulator/Nuclear Receptor Interaction by Ligand and DNA. Structure.

[135] Lai Y.-H., Choudhary K., Cloutier S.C., Xing Z., Aviran S., Tran E.J. (2019). Genome-Wide Discovery of DEAD-Box RNA Helicase Targets Reveals RNA Structural Remodeling in Transcription Termination. Genetics.

[136] Song C., Hotz-Wagenblatt A., Voit R., Grummt I. (2017). SIRT7 and the DEAD-Box Helicase DDX21 Cooperate to Resolve Genomic R Loops and Safeguard Genome Stability. Genes Dev..

[137] Taschuk F., Cherry S. (2020). DEAD-Box Helicases: Sensors, Regulators, and Effectors for Antiviral Defense. Viruses.

[138] Arconzo M., Piccinin E., Moschetta A. (2021). Increased Risk of Acute Liver Failure by Pain Killer Drugs in NAFLD: Focus on Nuclear Receptors and Their Coactivators. Dig. Liver Dis..

[139] Fornes D., Gomez Ribot D., Heinecke F., Roberti S.L., Capobianco E., Jawerbaum A. (2020). Maternal Diets Enriched in Olive Oil Regulate Lipid Metabolism and Levels of PPARs and Their Coactivators in the Fetal Liver in a Rat Model of Gestational Diabetes Mellitus. J. Nutr. Biochem..

[140] Kalliora C., Kyriazis I.D., Oka S.-I., Lieu M.J., Yue Y., Area-Gomez E., Pol C.J., Tian Y., Mizushima W., Chin A. (2019). Dual Peroxisome-Proliferator-Activated-Receptor-α/γ Activation Inhibits SIRT1-PGC1α Axis and Causes Cardiac Dysfunction. JCI Insight.

[141] Luo C., Widlund H.R., Puigserver P. (2016). PGC-1 Coactivators: Shepherding the Mitochondrial Biogenesis of Tumors. Trends Cancer.

[142] Stallcup M.R., Poulard C. (2020). Gene-Specific Actions of Transcriptional Coregulators Facilitate Physiological Plasticity: Evidence for a Physiological Coregulator Code. Trends Biochem. Sci..

[143] Emmett M.J., Lazar M.A. (2019). Integrative Regulation of Physiology by Histone Deacetylase 3. Nat. Rev. Mol. Cell Biol..

[144] Jaiswal B., Gupta A. (2018). Modulation of Nuclear Receptor Function by Chromatin Modifying Factor TIP60. Endocrinology.

[145] Jankowsky E., Guenther U.-P. (2019). A Helicase Links Upstream ORFs and RNA Structure. Curr. Genet..

[146] Surapureddi S., Viswakarma N., Yu S., Guo D., Rao M.S., Reddy J.K. (2006). PRIC320, a Transcription Coactivator, Isolated from Peroxisome Proliferator-Binding Protein Complex. Biochem. Biophys. Res. Commun..

[147] Jia Y., Liu N., Viswakarma N., Sun R., Schipma M.J., Shang M., Thorp E.B., Kanwar Y.S., Thimmapaya B., Reddy J.K. (2018). PIMT/NCOA6IP Deletion in the Mouse Heart Causes Delayed Cardiomyopathy Attributable to Perturbation in Energy Metabolism. Int. J. Mol. Sci..

[148] Jeronimo C., Robert F. (2017). The Mediator Complex: At the Nexus of RNA Polymerase II Transcription. Trends Cell Biol..

[149] Soutourina J. (2018). Transcription Regulation by the Mediator Complex. Nat. Rev. Mol. Cell Biol..

[150] Paiano A., Margiotta A., De Luca M., Bucci C. (2019). Yeast Two-Hybrid Assay to Identify Interacting Proteins. Curr. Protoc. Protein Sci..

[151] O’Malley B.W. (2016). Origins of the Field of Molecular Endocrinology: A Personal Perspective. Mol. Endocrinol..

[152] Kamei Y., Xu L., Heinzel T., Torchia J., Kurokawa R., Gloss B., Lin S.C., Heyman R.A., Rose D.W., Glass C.K. (1996). A CBP Integrator Complex Mediates Transcriptional Activation and AP-1 Inhibition by Nuclear Receptors. Cell.

[153] Sabari B.R., Dall’Agnese A., Boija A., Klein I.A., Coffey E.L., Shrinivas K., Abraham B.J., Hannett N.M., Zamudio A.V., Manteiga J.C. (2018). Coactivator Condensation at Super-Enhancers Links Phase Separation and Gene Control. Science.

[154] Tan H.W.S., Anjum B., Shen H.-M., Ghosh S., Yen P.M., Sinha R.A. (2019). Lysosomal Inhibition Attenuates Peroxisomal Gene Transcription via Suppression of PPARA and PPARGC1A Levels. Autophagy.

[155] Dumesic P.A., Egan D.F., Gut P., Tran M.T., Parisi A., Chatterjee N., Jedrychowski M., Paschini M., Kazak L., Wilensky S.E. (2019). An Evolutionarily Conserved UORF Regulates PGC1alpha and Oxidative Metabolism in Mice, Flies, and Bluefin Tuna. Cell Metab..

[156] Petr M., Stastny P., Zajac A., Tufano J.J., Maciejewska-Skrendo A. (2018). The Role of Peroxisome Proliferator-Activated Receptors and Their Transcriptional Coactivators Gene Variations in Human Trainability: A Systematic Review. Int. J. Mol. Sci..

[157] Behera A.K., Bhattacharya A., Vasudevan M., Kundu T.K. (2018). P53 Mediated Regulation of Coactivator Associated Arginine Methyltransferase 1 (CARM1) Expression Is Critical for Suppression of Adipogenesis. FEBS J..

[158] Xu W., Chen H., Du K., Asahara H., Tini M., Emerson B.M., Montminy M., Evans R.M. (2001). A Transcriptional Switch Mediated by Cofactor Methylation. Science.

[159] Kang Z., Fan R. (2020). PPARα and NCOR/SMRT Corepressor Network in Liver Metabolic Regulation. FASEB J..

[160] Ghisletti S., Huang W., Jepsen K., Benner C., Hardiman G., Rosenfeld M.G., Glass C.K. (2009). Cooperative NCoR/SMRT Interactions Establish a Corepressor-Based Strategy for Integration of Inflammatory and Anti-Inflammatory Signaling Pathways. Genes Dev..

[161] Jepsen K., Gleiberman A.S., Shi C., Simon D.I., Rosenfeld M.G. (2008). Cooperative Regulation in Development by SMRT and FOXP1. Genes Dev..

[162] Kumar S., Cunningham T.J., Duester G. (2016). Nuclear Receptor Corepressors Ncor1 and Ncor2 (Smrt) Are Required for Retinoic Acid-Dependent Repression of Fgf8 during Somitogenesis. Dev. Biol..

[163] Duong V., Augereau P., Badia E., Jalaguier S., Cavailles V. (2008). Regulation of Hormone Signaling by Nuclear Receptor Interacting Proteins. Adv. Exp. Med. Biol..

[164] Ogawa K., Yagi T., Guo T., Takeda K., Ohguchi H., Koyama H., Aotani D., Imaeda K., Kataoka H., Tanaka T. (2020). Pemafibrate, a Selective PPARα Modulator, and Fenofibrate Suppress Microglial Activation through Distinct PPARα and SIRT1-Dependent Pathways. Biochem. Biophys. Res. Commun..

[165] Purushotham A., Schug T.T., Xu Q., Surapureddi S., Guo X., Li X. (2009). Hepatocyte-Specific Deletion of SIRT1 Alters Fatty Acid Metabolism and Results in Hepatic Steatosis and Inflammation. Cell Metab..

[166] Han L., Zhou R., Niu J., McNutt M.A., Wang P., Tong T. (2010). SIRT1 Is Regulated by a PPAR{gamma}-SIRT1 Negative Feedback Loop Associated with Senescence. Nucleic Acids Res..

[167] Naiman S., Huynh F.K., Gil R., Glick Y., Shahar Y., Touitou N., Nahum L., Avivi M.Y., Roichman A., Kanfi Y. (2019). SIRT6 Promotes Hepatic Beta-Oxidation via Activation of PPARalpha. Cell Rep..

[168] Glass C.K., Rosenfeld M.G. (2000). The Coregulator Exchange in Transcriptional Functions of Nuclear Receptors. Genes Dev..

[169] Fritah A., Christian M., Parker M.G. (2010). The Metabolic Coregulator RIP140: An Update. Am. J. Physiol. Endocrinol. Metab..

[170] Venkata N.G., Robinson J.A., Cabot P.J., Davis B., Monteith G.R., Roberts-Thomson S.J. (2006). Mono(2-Ethylhexyl)Phthalate and Mono-n-Butyl Phthalate Activation of Peroxisome Proliferator Activated-Receptors Alpha and Gamma in Breast. Toxicol. Lett..

[171] Dawson M.I., Xia Z. (2012). The Retinoid X Receptors and Their Ligands. Biochim. Biophys. Acta.

[172] Brunmeir R., Xu F. (2018). Functional Regulation of PPARs through Post-Translational Modifications. Int. J. Mol. Sci..

[173] Iershov A., Nemazanyy I., Alkhoury C., Girard M., Barth E., Cagnard N., Montagner A., Chretien D., Rugarli E.I., Guillou H. (2019). The Class 3 PI3K Coordinates Autophagy and Mitochondrial Lipid Catabolism by Controlling Nuclear Receptor PPARα. Nat. Commun..

[174] Shalev A., Siegrist-Kaiser C.A., Yen P.M., Wahli W., Burger A.G., Chin W.W., Meier C.A. (1996). The Peroxisome Proliferator-Activated Receptor Alpha Is a Phosphoprotein: Regulation by Insulin. Endocrinology.

[175] Chamouton J., Latruffe N. (2012). PPARα/HNF4α Interplay on Diversified Responsive Elements. Relevance in the Regulation of Liver Peroxisomal Fatty Acid Catabolism. Curr. Drug Metab..

[176] Scarpulla R.C. (2011). Metabolic Control of Mitochondrial Biogenesis through the PGC-1 Family Regulatory Network. Biochim. Biophys. Acta.

[177] Hashimoto T. (1982). Individual Peroxisomal Beta-Oxidation Enzymes. Ann. N. Y. Acad. Sci..

[178] Reddy J.K. (2004). Peroxisome Proliferators and Peroxisome Proliferator-Activated Receptor Alpha: Biotic and Xenobiotic Sensing. Am. J. Pathol..

[179] Raas Q., Gondcaille C., Hamon Y., Leoni V., Caccia C., Menetrier F., Lizard G., Trompier D., Savary S. (2019). CRISPR/Cas9-Mediated Knockout of Abcd1 and Abcd2 Genes in BV-2 Cells: Novel Microglial Models for X-Linked Adrenoleukodystrophy. Biochim. Biophys. Acta Mol. Cell Biol. Lipids.

[180] Dixon E.D., Nardo A.D., Claudel T., Trauner M. (2021). The Role of Lipid Sensing Nuclear Receptors (PPARs and LXR) and Metabolic Lipases in Obesity, Diabetes and NAFLD. Genes.

[181] Wang Y., Nakajima T., Gonzalez F.J., Tanaka N. (2020). PPARs as Metabolic Regulators in the Liver: Lessons from Liver-Specific PPAR-Null Mice. Int. J. Mol. Sci..

[182] Haro D., Marrero P.F., Relat J. (2019). Nutritional Regulation of Gene Expression: Carbohydrate-, Fat- and Amino Acid-Dependent Modulation of Transcriptional Activity. Int. J. Mol. Sci..

[183] Vega R.B., Kelly D.P. (2017). Cardiac Nuclear Receptors: Architects of Mitochondrial Structure and Function. J. Clin. Investig..

[184] Gao Q., Jia Y., Yang G., Zhang X., Boddu P.C., Petersen B., Narsingam S., Zhu Y.-J., Thimmapaya B., Kanwar Y.S. (2015). PPARα-Deficient Ob/Ob Obese Mice Become More Obese and Manifest Severe Hepatic Steatosis Due to Decreased Fatty Acid Oxidation. Am. J. Pathol..

[185] Tonsgard J.H., Getz G.S. (1985). Effect of Reye’s Syndrome Serum on Isolated Chinchilla Liver Mitochondria. J. Clin. Investig..

[186] Inokuchi-Shimizu S., Park E.J., Roh Y.S., Yang L., Zhang B., Song J., Liang S., Pimienta M., Taniguchi K., Wu X. (2014). TAK1-Mediated Autophagy and Fatty Acid Oxidation Prevent Hepatosteatosis and Tumorigenesis. J. Clin. Investig..

[187] Park H.S., Lim J.H., Kim M.Y., Kim Y., Hong Y.A., Choi S.R., Chung S., Kim H.W., Choi B.S., Kim Y.S. (2016). Resveratrol Increases AdipoR1 and AdipoR2 Expression in Type 2 Diabetic Nephropathy. J. Transl. Med..

[188] Wanders R.J.A., Ferdinandusse S., Brites P., Kemp S. (2010). Peroxisomes, Lipid Metabolism and Lipotoxicity. Biochim. Biophys. Acta.

[189] Pontis S., Ribeiro A., Sasso O., Piomelli D. (2016). Macrophage-Derived Lipid Agonists of PPAR-α as Intrinsic Controllers of Inflammation. Crit. Rev. Biochem. Mol. Biol..

[190] Vluggens A., Andreoletti P., Viswakarma N., Jia Y., Matsumoto K., Kulik W., Khan M., Huang J., Guo D., Yu S. (2010). Reversal of Mouse Acyl-CoA Oxidase 1 (ACOX1) Null Phenotype by Human ACOX1b Isoform [Corrected]. Lab. Investig..

[191] Qi W., Gutierrez G.E., Gao X., Dixon H., McDonough J.A., Marini A.M., Fisher A.L. (2017). The ω-3 Fatty Acid α-Linolenic Acid Extends Caenorhabditis Elegans Lifespan via NHR-49/PPARα and Oxidation to Oxylipins. Aging Cell.

[192] Fock E., Lavrova E., Bachteeva V., Nikolaeva S., Parnova R. (2019). Suppression of Fatty Acid β-Oxidation and Energy Deficiency as a Cause of Inhibitory Effect of E. Coli Lipopolysaccharide on Osmotic Water Transport in the Frog Urinary Bladder. Comp. Biochem. Physiol. Part C Toxicol. Pharmacol..

[193] Ling Y., Shi Z., Yang X., Cai Z., Wang L., Wu X., Ye A., Jiang J. (2020). Hypolipidemic Effect of Pure Total Flavonoids from Peel of Citrus (PTFC) on Hamsters of Hyperlipidemia and Its Potential Mechanism. Exp. Gerontol..

[194] Li L.-Y., Lv H.-B., Jiang Z.-Y., Qiao F., Chen L.-Q., Zhang M.-L., Du Z.-Y. (2020). Peroxisomal Proliferator-Activated Receptor α-b Deficiency Induces the Reprogramming of Nutrient Metabolism in Zebrafish. J. Physiol..

[195] El Kebbaj Z., Andreoletti P., Mountassif D., Kabine M., Schohn H., Dauca M., Latruffe N., El Kebbaj M.S., Cherkaoui-Malki M. (2008). Differential Regulation of Peroxisome Proliferator-Activated Receptor (PPAR)-Alpha 1 and Truncated PPAR Alpha 2 as an Adaptive Response to Fasting in the Control of Hepatic Peroxisomal Fatty Acid Beta-Oxidation in the Hibernating Mammal. Endocrinology.

[196] Braissant O., Foufelle F., Scotto C., Dauça M., Wahli W. (1996). Differential Expression of Peroxisome Proliferator-Activated Receptors (PPARs): Tissue Distribution of PPAR-Alpha, -Beta, and -Gamma in the Adult Rat. Endocrinology.

[197] Warden A., Truitt J., Merriman M., Ponomareva O., Jameson K., Ferguson L.B., Mayfield R.D., Harris R.A. (2016). Localization of PPAR Isotypes in the Adult Mouse and Human Brain. Sci. Rep..

[198] Mariani M.M., Malm T., Lamb R., Jay T.R., Neilson L., Casali B., Medarametla L., Landreth G.E. (2017). Neuronally-Directed Effects of RXR Activation in a Mouse Model of Alzheimer’s Disease. Sci. Rep..

[199] Raso G.M., Esposito E., Vitiello S., Iacono A., Santoro A., D’Agostino G., Sasso O., Russo R., Piazza P.V., Calignano A. (2011). Palmitoylethanolamide Stimulation Induces Allopregnanolone Synthesis in C6 Cells and Primary Astrocytes: Involvement of Peroxisome-Proliferator Activated Receptor-α. J. Neuroendocrinol..

[200] Roy A., Jana M., Corbett G.T., Ramaswamy S., Kordower J.H., Gonzalez F.J., Pahan K. (2013). Regulation of Cyclic AMP Response Element Binding and Hippocampal Plasticity-Related Genes by Peroxisome Proliferator-Activated Receptor α. Cell Rep..

[201] Marx N., Duez H., Fruchart J.-C., Staels B. (2004). Peroxisome Proliferator-Activated Receptors and Atherogenesis: Regulators of Gene Expression in Vascular Cells. Circ. Res..

[202] Morinishi T., Tokuhara Y., Ohsaki H., Ibuki E., Kadota K., Hirakawa E. (2019). Activation and Expression of Peroxisome Proliferator-Activated Receptor Alpha Are Associated with Tumorigenesis in Colorectal Carcinoma. PPAR Res..

[203] Kliewer S.A., Forman B.M., Blumberg B., Ong E.S., Borgmeyer U., Mangelsdorf D.J., Umesono K., Evans R.M. (1994). Differential Expression and Activation of a Family of Murine Peroxisome Proliferator-Activated Receptors. Proc. Natl. Acad. Sci. USA.

[204] Costet P., Legendre C., Moré J., Edgar A., Galtier P., Pineau T. (1998). Peroxisome Proliferator-Activated Receptor Alpha-Isoform Deficiency Leads to Progressive Dyslipidemia with Sexually Dimorphic Obesity and Steatosis. J. Biol. Chem..

[205] Stec D.E., Gordon D.M., Hipp J.A., Hong S., Mitchell Z.L., Franco N.R., Robison J.W., Anderson C.D., Stec D.F., Hinds T.D. (2019). Loss of Hepatic PPARα Promotes Inflammation and Serum Hyperlipidemia in Diet-Induced Obesity. Am. J. Physiol. Regul. Integr. Comp. Physiol..

[206] Chen X., Ward S.C., Cederbaum A.I., Xiong H., Lu Y. (2017). Alcoholic Fatty Liver Is Enhanced in CYP2A5 Knockout Mice: The Role of the PPARα-FGF21 Axis. Toxicology.

[207] Francque S., Verrijken A., Caron S., Prawitt J., Paumelle R., Derudas B., Lefebvre P., Taskinen M.-R., Van Hul W., Mertens I. (2015). PPARα Gene Expression Correlates with Severity and Histological Treatment Response in Patients with Non-Alcoholic Steatohepatitis. J. Hepatol..

[208] Ip E., Farrell G.C., Robertson G., Hall P., Kirsch R., Leclercq I. (2003). Central Role of PPARalpha-Dependent Hepatic Lipid Turnover in Dietary Steatohepatitis in Mice. Hepatology.

[209] Patsouris D., Reddy J.K., Müller M., Kersten S. (2006). Peroxisome Proliferator-Activated Receptor Alpha Mediates the Effects of High-Fat Diet on Hepatic Gene Expression. Endocrinology.

[210] Stienstra R., Mandard S., Patsouris D., Maass C., Kersten S., Müller M. (2007). Peroxisome Proliferator-Activated Receptor Alpha Protects against Obesity-Induced Hepatic Inflammation. Endocrinology.

[211] Rando G., Tan C.K., Khaled N., Montagner A., Leuenberger N., Bertrand-Michel J., Paramalingam E., Guillou H., Wahli W. (2016). Glucocorticoid Receptor-PPARα Axis in Fetal Mouse Liver Prepares Neonates for Milk Lipid Catabolism. Elife.

[212] Montagner A., Polizzi A., Fouché E., Ducheix S., Lippi Y., Lasserre F., Barquissau V., Régnier M., Lukowicz C., Benhamed F. (2016). Liver PPARα Is Crucial for Whole-Body Fatty Acid Homeostasis and Is Protective against NAFLD. Gut.

[213] Polizzi A., Fouché E., Ducheix S., Lasserre F., Marmugi A.P., Mselli-Lakhal L., Loiseau N., Wahli W., Guillou H., Montagner A. (2016). Hepatic Fasting-Induced PPARα Activity Does Not Depend on Essential Fatty Acids. Int. J. Mol. Sci..

[214] Régnier M., Polizzi A., Lippi Y., Fouché E., Michel G., Lukowicz C., Smati S., Marrot A., Lasserre F., Naylies C. (2018). Insights into the Role of Hepatocyte PPARα Activity in Response to Fasting. Mol. Cell. Endocrinol..

[215] Brocker C.N., Patel D.P., Velenosi T.J., Kim D., Yan T., Yue J., Li G., Krausz K.W., Gonzalez F.J. (2018). Extrahepatic PPARα Modulates Fatty Acid Oxidation and Attenuates Fasting-Induced Hepatosteatosis in Mice. J. Lipid Res..

[216] Jordan S., Tung N., Casanova-Acebes M., Chang C., Cantoni C., Zhang D., Wirtz T.H., Naik S., Rose S.A., Brocker C.N. (2019). Dietary Intake Regulates the Circulating Inflammatory Monocyte Pool. Cell.

[217] Régnier M., Polizzi A., Smati S., Lukowicz C., Fougerat A., Lippi Y., Fouché E., Lasserre F., Naylies C., Bétoulières C. (2020). Hepatocyte-Specific Deletion of Pparα Promotes NAFLD in the Context of Obesity. Sci. Rep..

[218] Batatinha H.A.P., Lima E.A., Teixeira A.A.S., Souza C.O., Biondo L.A., Silveira L.S., Lira F.S., Rosa Neto J.C. (2017). Association Between Aerobic Exercise and Rosiglitazone Avoided the NAFLD and Liver Inflammation Exacerbated in PPAR-α Knockout Mice. J. Cell. Physiol..

[219] Brocker C.N., Yue J., Kim D., Qu A., Bonzo J.A., Gonzalez F.J. (2017). Hepatocyte-Specific PPARA Expression Exclusively Promotes Agonist-Induced Cell Proliferation without Influence from Nonparenchymal Cells. Am. J. Physiol. Gastrointest. Liver Physiol..

[220] Stanley W.C., Recchia F.A., Lopaschuk G.D. (2005). Myocardial Substrate Metabolism in the Normal and Failing Heart. Physiol. Rev..

[221] Kaimoto S., Hoshino A., Ariyoshi M., Okawa Y., Tateishi S., Ono K., Uchihashi M., Fukai K., Iwai-Kanai E., Matoba S. (2017). Activation of PPAR-α in the Early Stage of Heart Failure Maintained Myocardial Function and Energetics in Pressure-Overload Heart Failure. Am. J. Physiol. Heart Circ. Physiol..

[222] Di Paola R., Cordaro M., Crupi R., Siracusa R., Campolo M., Bruschetta G., Fusco R., Pugliatti P., Esposito E., Cuzzocrea S. (2016). Protective Effects of Ultramicronized Palmitoylethanolamide (PEA-Um) in Myocardial Ischaemia and Reperfusion Injury in VIVO. Shock.

[223] Standage S.W., Waworuntu R.L., Delaney M.A., Maskal S.M., Bennion B.G., Duffield J.S., Parks W.C., Liles W.C., McGuire J.K. (2016). Nonhematopoietic Peroxisome Proliferator-Activated Receptor-α Protects Against Cardiac Injury and Enhances Survival in Experimental Polymicrobial Sepsis. Crit. Care Med..

[224] Yammine A., Namsi A., Vervandier-Fasseur D., Mackrill J.J., Lizard G., Latruffe N. (2021). Polyphenols of the Mediterranean Diet and Their Metabolites in the Prevention of Colorectal Cancer. Molecules.

[225] Castrejón-Tellez V., Rodríguez-Pérez J.M., Pérez-Torres I., Pérez-Hernández N., Cruz-Lagunas A., Guarner-Lans V., Vargas-Alarcón G., Rubio-Ruiz M.E. (2016). The Effect of Resveratrol and Quercetin Treatment on PPAR Mediated Uncoupling Protein (UCP-) 1, 2, and 3 Expression in Visceral White Adipose Tissue from Metabolic Syndrome Rats. Int. J. Mol. Sci..

[226] Zhou Y., Lin S., Zhang L., Li Y. (2016). Resveratrol Prevents Renal Lipotoxicity in High-Fat Diet-Treated Mouse Model through Regulating PPAR-α Pathway. Mol. Cell. Biochem..

[227] Barone R., Rizzo R., Tabbì G., Malaguarnera M., Frye R.E., Bastin J. (2019). Nuclear Peroxisome Proliferator-Activated Receptors (PPARs) as Therapeutic Targets of Resveratrol for Autism Spectrum Disorder. Int. J. Mol. Sci..

[228] Fantacuzzi M., De Filippis B., Amoroso R., Giampietro L. (2019). PPAR Ligands Containing Stilbene Scaffold. Mini Rev. Med. Chem..

[229] Bastin J., Djouadi F. (2016). Resveratrol and Myopathy. Nutrients.

[230] Sun X., Yamasaki M., Katsube T., Shiwaku K. (2015). Effects of Quercetin Derivatives from Mulberry Leaves: Improved Gene Expression Related Hepatic Lipid and Glucose Metabolism in Short-Term High-Fat Fed Mice. Nutr. Res. Pract..

[231] Wang L.L., Zhang Z.C., Hassan W., Li Y., Liu J., Shang J. (2016). Amelioration of Free Fatty Acid-Induced Fatty Liver by Quercetin-3-O-β-D-Glucuronide through Modulation of Peroxisome Proliferator-Activated Receptor-Alpha/Sterol Regulatory Element-Binding Protein-1c Signaling. Hepatol. Res..

[232] Chen D., Daniel K.G., Kuhn D.J., Kazi A., Bhuiyan M., Li L., Wang Z., Wan S.B., Lam W.H., Chan T.H. (2004). Green Tea and Tea Polyphenols in Cancer Prevention. Front. Biosci..

[233] Zhang S., Yang X., Luo J., Ge X., Sun W., Zhu H., Zhang W., Cao J., Hou Y. (2014). PPARα Activation Sensitizes Cancer Cells to Epigallocatechin-3-Gallate (EGCG) Treatment via Suppressing Heme Oxygenase-1. Nutr. Cancer.

[234] Yang H., Zuo X.Z., Tian C., He D.L., Yi W.J., Chen Z., Zhang P.W., Ding S.B., Ying C.J. (2015). Green Tea Polyphenols Attenuate High-Fat Diet-Induced Renal Oxidative Stress through SIRT3-Dependent Deacetylation. Biomed. Environ. Sci..

[235] Chen J.-W., Kong Z.-L., Tsai M.-L., Lo C.-Y., Ho C.-T., Lai C.-S. (2018). Tetrahydrocurcumin Ameliorates Free Fatty Acid-Induced Hepatic Steatosis and Improves Insulin Resistance in HepG2 Cells. J. Food Drug Anal..

[236] Rimando A.M., Khan S.I., Mizuno C.S., Ren G., Mathews S.T., Kim H., Yokoyama W. (2016). Evaluation of PPARα Activation by Known Blueberry Constituents. J. Sci. Food Agric..

[237] Vitaglione P., Mazzone G., Lembo V., D’Argenio G., Rossi A., Guido M., Savoia M., Salomone F., Mennella I., De Filippis F. (2019). Coffee Prevents Fatty Liver Disease Induced by a High-Fat Diet by Modulating Pathways of the Gut-Liver Axis. J. Nutr. Sci..

[238] Bigagli E., Toti S., Lodovici M., Giovannelli L., Cinci L., D’Ambrosio M., Luceri C. (2018). Dietary Extra-Virgin Olive Oil Polyphenols Do Not Attenuate Colon Inflammation in Transgenic HLAB-27 Rats but Exert Hypocholesterolemic Effects through the Modulation of HMGCR and PPAR-α Gene Expression in the Liver. Lifestyle Genom..

[239] Pirozzi C., Lama A., Simeoli R., Paciello O., Pagano T.B., Mollica M.P., Di Guida F., Russo R., Magliocca S., Canani R.B. (2016). Hydroxytyrosol Prevents Metabolic Impairment Reducing Hepatic Inflammation and Restoring Duodenal Integrity in a Rat Model of NAFLD. J. Nutr. Biochem..

[240] Valenzuela R., Illesca P., Echeverría F., Espinosa A., Rincón-Cervera M.Á., Ortiz M., Hernandez-Rodas M.C., Valenzuela A., Videla L.A. (2017). Molecular Adaptations Underlying the Beneficial Effects of Hydroxytyrosol in the Pathogenic Alterations Induced by a High-Fat Diet in Mouse Liver: PPAR-α and Nrf2 Activation, and NF-ΚB down-Regulation. Food Funct..

[241] El Kebbaj R., Andreoletti P., El Hajj H.I., El Kharrassi Y., Vamecq J., Mandard S., Saih F.-E., Latruffe N., El Kebbaj M.S., Lizard G. (2015). Argan Oil Prevents Down-Regulation Induced by Endotoxin on Liver Fatty Acid Oxidation and Gluconeogenesis and on Peroxisome Proliferator-Activated Receptor Gamma Coactivator-1α, (PGC-1α), Peroxisome Proliferator-Activated Receptor α (PPARα) and Estrogen Related Receptor α (ERRα). Biochim. Open.

[242] Chen X., Wang Q., Shao M., Ma L., Guo D., Wu Y., Gao P., Wang X., Li W., Li C. (2019). Ginsenoside Rb3 Regulates Energy Metabolism and Apoptosis in Cardiomyocytes via Activating PPARα Pathway. Biomed. Pharmacother..

[243] Zhang C., Deng J., Liu D., Tuo X., Xiao L., Lai B., Yao Q., Liu J., Yang H., Wang N. (2018). Nuciferine Ameliorates Hepatic Steatosis in High-Fat Diet/Streptozocin-Induced Diabetic Mice through a PPARα/PPARγ Coactivator-1α Pathway. Br. J. Pharmacol..

[244] Yu H., Li C., Yang J., Zhang T., Zhou Q. (2016). Berberine Is a Potent Agonist of Peroxisome Proliferator Activated Receptor Alpha. Front. Biosci..

